# Dietary Polyphenols as Adjunctive Therapy for Inflammatory Bowel Disease: A Systematic Review and Meta-Analysis of Randomized Controlled Trials

**DOI:** 10.3390/nu18203335

**Published:** 2026-10-12

**Authors:** Yuang Dong, Huitong Xu, Dongyang Zhang, Xinxin Ren, Xinyu Wu, Zhou Zheng, Qingsheng Yu, Yi Shen

**Affiliations:** 1 The First Clinical Medical College, Anhui University of Chinese Medicine, Hefei 230012, China; 17855992638@163.com (Y.D.); 15552615112@163.com (H.X.); zdy1024744154@163.com (D.Z.); 19855117037@163.com (X.W.);; 2 College of Integrated Chinese and Western Medicine, Anhui University of Chinese Medicine, Hefei 230012, China; 15249820278@163.com

**Keywords:** inflammatory bowel disease, ulcerative colitis, Crohn’s disease, dietary polyphenols, curcumin, meta-analysis

## Abstract

Background: Dietary polyphenols have been studied as adjunctive therapies for inflammatory bowel disease (IBD), but trials vary in formulation, clinical setting, and outcome definitions. We evaluated efficacy and safety separately for ulcerative colitis (UC) and Crohn’s disease (CD). Methods: We searched MEDLINE/PubMed, China National Knowledge Infrastructure (CNKI), Wanfang, Web of Science, Embase, Cochrane Library, and ClinicalTrials.gov through 20 August 2026. Risk of bias was assessed using Cochrane Risk of Bias 2 (RoB 2). Meta-analyses used MDs, SMDs, or ORs with 95% CIs. Quantitative synthesis was undertaken when studies were sufficiently comparable in clinical setting, outcome definition, and data structure; otherwise, results were summarized using study-level estimates. The protocol was registered with PROSPERO (CRD420261451126). Results: Twenty-one reports representing 19 randomized trials and 1048 participants were included. In conventional UC, polyphenols were associated with lower SCCAI scores (MD −1.32, 95% CI −1.81 to −0.83; *I*^2^ = 0%), whereas CRP/hs-CRP did not show a clear pooled effect (SMD 0.29, 95% CI −0.32 to 0.90; *I*^2^ = 83.7%). IBDQ-9 improved (MD 5.57, 95% CI 2.88 to 8.26). Clinical response favored supplementation (OR 4.90, 95% CI 3.04 to 7.89), as did induction of clinical remission (OR 5.98, 95% CI 3.08 to 11.59; *I*^2^ = 22.6%). Supplementation also favored endoscopic remission (OR 10.81, 95% CI 1.67 to 69.93; *I*^2^ = 69.4%), but the estimate was imprecise and sensitive to study-level risk of bias. In CD, the two randomized trials were not pooled due to targeting different clinical settings. Safety reporting was heterogeneous and sparse, with no consistent pattern of increased harm across trials. Conclusions: Curcumin has the most extensive adjunctive evidence base in UC but effect estimates for several outcomes were imprecise or sensitive to study design. Evidence in CD is still lacking.

## 1. Introduction

Ulcerative colitis (UC) and Crohn’s disease (CD) are usually considered together as inflammatory bowel disease (IBD), but they may be biologically different in subtle ways. Both have a relapsing course and neither can be explained by a single defect [1,2]. There is an inherited susceptibility, as well as microbial imbalance, compromised mucosal defenses, and failure of the immune response to resolve normally. These are not distinct causes but interacting processes [3,4,5]. Epidemiology has also grown beyond its traditional geographic boundaries. Population studies and Global Burden of Disease estimates indicate a rapid increase in incidence in the industrializing regions of Asia, Latin America and Africa [6,7,8]. IBD is thus increasingly not a Western disease. The impact of this change will accumulate over time: a relapsing disease adds to both prevalence and lifelong treatment exposure and social cost.

The same dependence can be seen in the inflamed gut. Epithelium, immunity, microbes and oxidative stress cannot be separated into discrete parts of the disease process [9]. This results in increased permeability of epithelial surface and increased access of luminal antigens and microbial material to mucosa. Innate and adaptive immune responses may then persist rather than subside [9,10]. Oxidative injury adds to the damage. Reactive oxygen species (ROS) oxidize lipids and proteins, injure DNA, promote epithelial-cell death, and weaken tight junctions [11]. Sustained nuclear factor kappa B (NF-κB) signaling, coupled with an inadequate nuclear factor erythroid 2-related factor 2 (Nrf2) response, allows inflammatory and oxidative processes to reinforce one another [4,11,12]. The microbiota is involved at several points in this sequence. Its metabolites affect epithelial repair and immune-cell differentiation, as well as the release of inflammatory mediators [13]. Dysbiosis is thus woven into the disease process rather than sitting alongside it as an unrelated abnormality.

Biologics and targeted small molecules have changed the view of moderate-to-severe IBD [14]. However, some patients never respond, while others recover initially but then lose it. The risk of infection or cost may also restrict continued treatment [15,16,17]. Repairing epithelial leakage, correcting dysbiosis or arresting oxidative damage may not be achieved by blocking a single immune target. This leaves a rationale to study treatments with broader actions so long as they are used as adjuncts to established pharmacological care and not as a replacement.

Polyphenols are molecules derived from plants, but the reason they are relevant here is not that they are derived from plants, but that they may have pharmacological effects. Radical scavenging and modulation of NF-κB and Nrf2 signaling has been reported in experimental studies [11,12,18]. Other effects have been described on microbial metabolism and on the integrity of epithelial junctions. Therefore, early clinical trials have been initiated with curcumin, resveratrol and other such products, with investigators reporting changes in activity scores, inflammatory parameters or mucosal outcomes [19,20,21,22].

The clinical literature is not consistent at this point. The compounds have different absorption and different exposure in the colon. Trial formulations and doses differ. Background medications and treatment periods differ. Most studies have small sample sizes. These differences make it difficult to determine whether an observed effect is due to a specific product or polyphenols in general. They also fail to resolve two practical uncertainties: whether the results of UC are applicable to CD and whether the effects observed during induction are sustained in a maintenance setting.

As a result, we did not assume a class effect when synthesizing the randomized evidence. We considered efficacy and adverse events, separating UC and CD whenever possible, and clinical heterogeneity in the interpretation, which is not to be overlooked.

## 2. Materials and Methods

### 2.1. Protocol and Registration

This systematic review and meta-analysis was registered in PROSPERO (CRD420261451126) and adhered to the PRISMA 2020 statement for conduct and reporting. The registration entry was established on 14 July 2026, prior to the conclusion of the final database inquiry on 20 August 2026; it was later revised and most recently modified on 26 September 2026. September update added a section on reviewer responsibilities. Studies were independently screened, data extracted and risk of bias assessed by Huitong Xu and Yuang Dong; Dongyang Zhang was a third reviewer when no consensus could be reached. The update also specified sensitivity handling for the silymarin trial of Rastegarpanah et al. because of a discrepancy between the *p* value reported in the publication and the remission counts reported. Induction and maintenance trials were analyzed separately. The Rastegarpanah trial provides maintenance evidence and is presented with the published completer analysis and a conservative intent-to-treat sensitivity analysis. These amendments clarified methods and reporting. The review question, eligibility criteria, primary outcomes and overall analytical framework were unchanged.

### 2.2. Inclusion and Exclusion Criteria

#### 2.2.1. Study Population

Eligibility required a confirmed diagnosis of ulcerative colitis or Crohn’s disease. Exclusion criteria were not defined by sex, age, ethnicity or geographical setting.

#### 2.2.2. Intervention and Control

Intervention arms received a defined polyphenol supplement either alone or with usual treatment. Eligible products included isolated compounds and plant preparations with a stated polyphenol content. Comparators were placebo, usual care, or the same background regimen without the polyphenol.

#### 2.2.3. Outcome Measures

A trial had to report at least one prespecified endpoint. Primary outcomes were disease activity scores, endoscopic remission, clinical response and clinical remission. Secondary endpoints were hemoglobin, quality of life, adverse events and inflammatory or oxidative stress biomarkers. The instruments used were total or partial Mayo scores, Crohn’s Disease Activity Index (CDAI), Simple Clinical Colitis Activity Index (SCCAI), fecal calprotectin, C-reactive protein/high-sensitivity C-reactive protein (CRP/hs-CRP), tumor necrosis factor alpha (TNF-α), erythrocyte sedimentation rate (ESR), total antioxidant capacity (TAC), malondialdehyde (MDA), and Inflammatory Bowel Disease Questionnaire (IBDQ).

#### 2.2.4. Design of the Study

Randomized controlled trials (RCTs) of a dietary polyphenol intervention in IBD were eligible. There was no restriction on date or language.

#### 2.2.5. Criteria of Exclusion

We excluded animal studies, reviews, case reports, conference abstracts and other non-original reports. Studies were also excluded if the polyphenol composition or dose could not be identified, participants did not have IBD, IBD-specific data were not available, essential outcome data could not be retrieved or the report was a duplicate of an included trial.

### 2.3. Literature Search Strategy

The Cochrane Library, PubMed, MEDLINE Complete, Embase, Web of Science, Wanfang, ClinicalTrials.gov, and China National Knowledge Infrastructure (CNKI) were searched from inception to 20 August 2026, without language restrictions. The full search strategies are given in Supplementary Table S1. Before screening, all of the records that were found were put into a database for managing references. If a DOI or PMID was available, it was used to find duplicates first. Then, normalized titles, authors, publication year, journal, volume, and page or article numbers were compared to find any remaining duplicates. These were checked by hand after automated matching. For articles retrieved from several databases, one bibliographic record was kept. Companion publications from the same randomized trial were retained as separate reports and linked at the trial level during data extraction.

### 2.4. Literature Screening, Data Extraction, and Assessment of Risk of Bias

The risk-of-bias assessment, data extraction, and study screening were conducted independently by two reviewers, Huitong Xu (HX) and Yuang Dong (YD). Initial screening included titles and abstracts, and then full-text evaluation of reports that might be eligible. Disagreements were resolved through discussion; when consensus could not be reached, Dongyang Zhang (DZ) was brought in as a third reviewer. The extraction form included information on the study population, intervention, comparator, follow-up, outcomes, and items required for risk-of-bias assessment. HX and YD independently assessed risk of bias using the Cochrane Risk of Bias 2 (RoB 2) tool for randomized trials, with unresolved disagreements adjudicated by DZ.

RoB 2 assessments focused on the effect of assignment to intervention (the intention-to-treat effect) for each specific result contributing to the review. Five domains were assessed: the randomization process, deviations from intended interventions, missing outcome data, outcome measurement, and selection of the reported result. Signaling questions and the RoB 2 algorithm guided judgments for each domain and for the result overall, using the categories low risk, some concerns, or high risk of bias [23,24,25]. Companion publications were linked to the same underlying randomized trial when their enrollment periods, participant profiles, intervention schedules, sample sizes, and registration details indicated a common cohort. These reports were not counted as independent trials in any pooled analysis.

### 2.5. Statistical Analysis

Analyses were conducted in Review Manager (RevMan) 5.3 [26]. Continuous outcomes were expressed as mean differences (MDs) when measured on the same scale, or standardized mean differences (SMDs) when the same construct was measured on materially different numerical scales; dichotomous outcomes were expressed as odds ratios (ORs). Study-reported means and standard deviations (SDs) were used wherever possible. Baseline characteristics presented as medians and interquartile ranges (IQRs) were kept in that form when they were not used to estimate effects. For outcomes reported only as medians with an IQR or range, means were estimated using Luo et al. [27] and SDs using Wan et al. [28], provided sufficient summary statistics were available. Outcomes were excluded from mean-based pooling when the published data did not support a reliable conversion. Final post-intervention values and changes from baseline were pooled in the same MD analysis only when they represented a common, clinically interpretable estimand.

Study number alone did not determine whether results were pooled. Clinical and methodological comparability was assessed by considering polyphenol type and formulation, dose, disease phenotype and clinical setting, background therapy, outcome definition, follow-up duration, and analysis set. Pooling proceeded only when the studies were similar enough to yield a clinically meaningful combined estimate; where differences in clinical setting, outcome definition, or data structure made that estimate difficult to interpret, individual-study effects were reported or findings were summarized descriptively. Statistical heterogeneity was described using Cochran’s Q and *I*^2^. A fixed-effect model was used when a common underlying treatment effect appeared plausible, and a random-effects model when the true effect was more likely to vary across studies.

For sparse binary outcomes, Mantel–Haenszel ORs were used for the principal analyses. A continuity correction of 0.5 was applied when required for a single zero cell, whereas studies with zero events in both groups did not contribute to a relative-effect estimate. Because sparse-event estimates may be sensitive to continuity correction, an alternative risk-difference analysis was performed as a sensitivity analysis for the curcumin endoscopic-remission outcome. All effect estimates are reported with 95% confidence intervals (CIs) and two-sided *p* values. In multi-arm trials, shared control groups were counted only once within a given analysis. When two relevant intervention arms represented the same comparison class, the active arms were combined before comparison with the shared control.

Studies were grouped by polyphenol type, disease phenotype, and clinical setting. Induction in active disease, maintenance of remission, prevention of postoperative recurrence, and post-IPAA pouchitis were considered separately. A trial reporting different eligible outcomes could contribute to more than one prespecified analytical perspective, although each trial was counted only once within a given pooled analysis. Limitations in certainty were examined through outcome-specific RoB 2 judgments, statistical inconsistency, imprecision, indirectness due to clinical heterogeneity, and sensitivity analyses. Supplementary Table S2 presents the trial-level RoB 2 domain judgments and the reasons for the missing-outcome-data assessments; Supplementary Table S3 summarizes sensitivity analyses excluding results judged at high risk of bias in Domain 3.

## 3. Results

### 3.1. Search Results

The searches identified 1377 records. After removal of 533 duplicates, 844 records were screened by title and abstract, of which 814 were excluded. The full texts of 30 reports were assessed for eligibility, and nine reports were excluded. Twenty-one reports representing 19 independent randomized trials involving 1048 participants were included in the review [19,20,21,22,29,30,31,32,33,34,35,36,37,38,39,40,41,42,43,44,45]. The study selection process is shown in Figure 1.

### 3.2. Risk of Bias Assessment

Risk of bias was assessed using the Cochrane RoB 2 framework. After companion publications were linked to their underlying randomized trials, 19 independent trials were represented. At the trial level, the overall judgment was low risk in 4/19 trials (21.1%), some concerns in 9/19 (47.4%), and high risk in 6/19 (31.6%). High overall risk was driven primarily by Domain 3 (missing outcome data), particularly in trials that relied on per-protocol or completer analyses after appreciable or potentially outcome-related attrition. Because RoB 2 is result-specific, outcome-specific differences between companion reports are retained in Supplementary Table S2. Figure 2 summarizes the distribution of domain-level judgments across evaluated results. Sensitivity analyses excluding Domain 3 high-risk results are summarized in Supplementary Table S3. SCCAI remained robust after exclusion of Nikkhah-Bodaghi (MD = −1.28, 95% CI −1.82 to −0.73; *I*^2^ = 0%), and CRP/hs-CRP remained null after exclusion of Amiri and Langmead (SMD = −0.02, 95% CI −0.47 to 0.44). Clinical-remission estimates also remained favorable after excluding Biedermann and Langmead, whereas endoscopic-remission estimates retained their direction but changed substantially in magnitude after exclusion of Biedermann. These sensitivity analyses indicate that the principal clinical-remission conclusions were not driven solely by studies at high risk because of missing outcome data, while the magnitude of the endoscopic effect remains less stable.

### 3.3. Characteristics of the Included Trials

Seven polyphenol interventions were represented, including resveratrol, curcumin, anthocyanins, silymarin, and three polyphenol-rich plant products (ginger, aloe vera gel, and *Achillea wilhelmsii*). The 21 included reports corresponded to 19 independent randomized trials involving 1048 randomized or enrolled participants at the trial level. Sixteen reports enrolled patients with conventional UC only, two enrolled patients with CD only, two included both UC and CD populations without separate disease-specific results, and one study evaluated a distinct post-IPAA pouchitis population [39]. The latter study was retained in the systematic review but was considered separately from conventional UC and CD in the quantitative synthesis.

The Samsamikor publications from 2015 and 2016 originated from the same resveratrol trial [21,29], while the Martins publications from 2024 and 2025 arose from the same registered three-arm trial [31,32]. These companion reports were therefore counted once at the trial level. Randomized sample sizes for each independent trial are presented in Table 1. Because attrition, missing outcome data, and per-protocol or available-case analyses resulted in smaller analysis populations for some outcomes, the exact denominators used in individual meta-analyses are reported in the corresponding forest plots.

The later meta-analyses grouped studies by both polyphenol type and disease phenotype, so some trials and participants appeared in more than one analytical grouping. This was especially common among curcumin trials in UC: when the relevant outcome was reported, the same trial could contribute to the curcumin-specific analysis in later results, the overall IBD analysis in the Results section, and the UC-specific analyses in later sections. These analyses provide complementary viewsof a substantially overlapping evidence base rather than independent sets of evidence. Within any single pooled analysis, each eligible trial was included only once.

### 3.4. Curcumin-Specific Evidence

#### 3.4.1. Key Clinical Outcomes

Six conventional curcumin trials reported clinical response in 161 participants receiving curcumin and 144 controls. Heterogeneity was low (*I*^2^ = 17%, *p* = 0.30), and fixed-effect Mantel–Haenszel pooling found higher odds of response with curcumin (OR = 5.39, 95% CI 3.20 to 9.09, *p* < 0.00001; Figure 3A). Seven trials in active disease assessed remission during induction; the random-effects estimate favored curcumin among 181 recipients and 154 controls (OR = 7.36, 95% CI 3.75 to 14.44; *I*^2^ = 13.5%; Figure 3B). Maintenance, postoperative, and post-IPAA trials were considered separately because their clinical contexts differed. Endoscopic remission data came from four trials, with 95 intervention participants and 79 controls. Fixed-effect Mantel–Haenszel pooling gave an OR of 18.13 (95% CI 6.03 to 54.51; Figure 3C), while an alternative random-effects analysis also favored curcumin when expressed as a risk difference (RD = 0.43, 95% CI 0.17 to 0.68). Heterogeneity in the latter analysis was high (*I*^2^ = 85%), leaving the size of the endoscopic effect uncertain.

#### 3.4.2. Secondary Curcumin-Specific Outcomes

Curcumin was associated with lower SCCAI scores in three trials reporting post-intervention means (MD = −1.29, 95% CI −1.90 to −0.69; *I*^2^ = 0%; Supplementary Figure S1A). Lang et al. could not be included in this continuous-outcome analysis because the article did not report an end-of-treatment SCCAI mean ± SD. The three-trial CRP/hs-CRP estimate showed no clear effect (SMD = 0.06, 95% CI −0.49 to 0.61; *I*^2^ = 60.0%; Supplementary Figure S1B). Two trials provided ESR and TNF-α data; study-specific estimates are shown in Supplementary Figure S1C,D. Post-treatment ESR differences were −6.04 mm/h in Sadeghi et al. (95% CI −12.81 to 0.73) and 3.90 mm/h in Ebrahimi Daryani et al. (95% CI −1.63 to 9.43); the corresponding TNF-α SMDs were −0.27 (95% CI −0.74 to 0.21) and 0.41 (95% CI −0.24 to 1.06). The Mayo outcomes also remained separate: Ebrahimi Daryani reported the post-treatment total Mayo score, while Lang reported change in the endoscopic Mayo subscore. Recurrence results were reported by clinical setting, with UC maintenance relapse from Hanai considered separately from CD postoperative recurrence in Bommelaer.

#### 3.4.3. Post-IPAA Pouchitis

He et al. evaluated curcumin in a clinically distinct population of patients who had undergone total proctocolectomy with ileal pouch-anal anastomosis (IPAA) [39]. The study included 52 participants, comprising a maintenance cohort of 39 patients without active pouchitis and an induction cohort of 13 patients with chronic refractory pouchitis. All participants received concomitant probiotic therapy in addition to curcumin or placebo, and disease activity was assessed using the Pouchitis Disease Activity Index (PDAI) rather than conventional UC activity indices.

In the maintenance cohort, 17 of 19 patients receiving curcumin met the study-defined clinical-remission criteria at the end of treatment, compared with 1 of 20 patients receiving placebo. In the induction cohort, four of six curcumin-treated patients achieved a clinical response, compared with none of the seven placebo-treated patients; however, no patient in the induction cohort achieved the study-defined clinical-remission criteria. Because this post-IPAA population, concomitant treatment, and outcome framework differed substantially from those of conventional UC trials, the study was retained in the systematic review but was not included in the pooled curcumin-specific, UC-specific, or overall IBD clinical-response/remission analyses.

### 3.5. Results on the Efficacy of Resveratrol

Two randomized trials reported CRP/hs-CRP after resveratrol supplementation. Samsami-kor et al. found post-treatment hs-CRP levels of 2.58 ± 1.79 mg/L with resveratrol and 3.54 ± 2.35 mg/L in controls (SMD = −0.45, 95% CI −1.02 to 0.12). In Erol Dogan et al., CRP was 3.60 ± 1.50 mg/L with a Mediterranean diet plus resveratrol, compared with 3.20 ± 0.10 mg/L with the diet alone (SMD = 0.37, 95% CI −0.33 to 1.07). The estimates pointed in opposite directions, and both confidence intervals included zero. These trials do not show a consistent reduction in circulating CRP/hs-CRP with resveratrol supplementation.

### 3.6. Results on the Efficacy of Ginger

For ginger, the review had only one placebo-controlled trial, a 12-week study in which 46 patients with mild-to-moderately active UC completed treatment, 22 on 2000 mg of dried ginger powder per day and 24 on placebo [41]; baseline MDA was 8.33 ± 1.82 and 7.88 ± 2.24 in the two groups, respectively (*p* = 0.348), but the values had separated by week 6 (5.54 ± 2.03 versus 7.02 ± 0.87; *p* = 0.003) and were farther apart at week 12 (3.87 ± 1.95 versus 6.38 ± 2.42; *p* < 0.001; Figure 4A), whereas TAC at the final visit was almost identical (2.16 ± 1.16 versus 2.17 ± 1.17; *p* = 0.640; Figure 4B). At week 12, Simple Clinical Colitis Activity Index Questionnaire (SCCAIQ) had fallen from 7.60 ± 4.03 to 4.05 ± 1.23 with ginger and from 6.20 ± 3.22 to 5.55 ± 2.39 with placebo (*p* = 0.017; Figure 4C); the 9-item Inflammatory Bowel Disease Questionnaire (IBDQ-9) score moved from 44.22 ± 9.79 to 47.23 ± 9.24 in the ginger arm and from 43.12 ± 6.00 to 41.87 ± 14.18 under placebo, although this comparison was not significant (*p* = 0.140; Figure 4D), so this small trial provides preliminary evidence for lower lipid peroxidation and disease activity but not for improved TAC or overall quality of life.

### 3.7. Results on the Efficacy of Anthocyanins

The only randomized evidence for anthocyanins in active UC was obtained in a multicentre phase IIa trial [35]. Recruitment was interrupted due to coronavirus disease 2019 (COVID-19) before the planned sample size of 100; 34 patients were randomized and the per-protocol analysis included 18 ACRE and eight placebo subjects. The primary endpoint was not achieved. At week 8, the clinical response rate was 50.0% (9/18 participants) in the anthocyanin-rich extract (ACRE) group and 37.5% (3/8 participants) in the placebo group (OR, 1.667; 90% CI, 0.399 to 6.963; one-sided *p* = 0.278). Remission of symptoms was seen in 44.4% vs. 37.5% of participants and 72.2% vs. 50.0% had no rectal bleeding. Endoscopic remission rates were 22.2% and 25.0%, respectively. No statistically significant differences were observed between the groups for any of these outcomes (Figure 5A). Calprotectin decreased from 1049 ± 1139 to 557 ± 756 μg/g with ACRE, whereas it increased from 947 ± 1039 to 1040 ± 1179 μg/g with placebo (*p* = 0.035 for the between-group change; Figure 5B). The Mayo score decreased by 2.61 ± 2.79 with ACRE and by 2.00 ± 3.07 with placebo, with no significant between-group difference (Figure 5C). The biomarker finding is interesting, but the prematurely terminated, underpowered trial does not establish clinical efficacy.

### 3.8. Results on the Efficacy of Aloe Vera Gel

A 4-week double-blind RCT randomized 44 evaluable patients with mild-to-moderate active UC in a 2:1 ratio to oral aloe vera gel (*n* = 30) or placebo (*n* = 14) [40]. Clinical remission occurred in 9/30 patients receiving aloe vera (30%) and 1/14 receiving placebo (7%; OR = 5.6, 95% CI 0.6 to 49, *p* = 0.09). Clinical improvement was reported in 11/30 versus 1/14 patients (37% versus 7%; OR = 7.5, 95% CI 0.9 to 66, *p* = 0.06), while clinical response occurred in 14/30 versus 2/14 (47% versus 14%; OR = 5.3, 95% CI 1.0 to 27, *p* = 0.048). Only clinical response reached the significance threshold (Figure 6). The ORs for sigmoidoscopic remission and improvement were 1.7 and 2.6, respectively; those for histological remission and improvement were 0.5 and 1.2. These estimates had wide confidence intervals that crossed 1. SCCAI and histology improved from baseline in the aloe vera group, but sigmoidoscopy, hemoglobin, platelets, ESR, CRP, and albumin showed no clear advantage over placebo. The findings suggest a possible short-term symptomatic response, although endoscopic and histological remission benefits remain unproven.

### 3.9. Efficacy Results for Achillea wilhelmsii

In a 4-week, double-blind study, 49 patients with mild-to-moderate active UC were randomized to *Achillea wilhelmsii* (*n* = 23) or placebo (*n* = 26), and 20 participants in each group completed treatment [43]. The partial Mayo score decreased from 5.35 ± 1.69 at baseline to 2.45 ± 2.16 at week 4 in the Achillea group and from 5.00 ± 1.94 to 2.65 ± 1.89 in the placebo group. Both groups showed significant within-group improvement (*p* < 0.001), but the between-group difference in improvement was not significant (*p* = 1.000; Figure 7A). Improvement in stool frequency occurred in 11/20 versus 16/20 participants (*p* = 0.176), rectal bleeding in 10/20 versus 13/20 (*p* = 0.523), physician-rated activity in 13/20 versus 9/20 (*p* = 0.341), and partial Mayo classification in 17/20 versus 17/20 (*p* = 1.000; Figure 7B). Hemoglobin, platelets, ESR, and CRP did not differ clearly between groups. One participant receiving Achillea developed grade 1 contact dermatitis that resolved after withdrawal. The study suggests short-term tolerability but no clear efficacy beyond placebo.

### 3.10. Efficacy Results for Silymarin

A randomized, double-blind, placebo-controlled trial assessed silymarin for maintaining remission in UC. Of 80 participants randomized to silymarin (*n* = 42) or placebo (*n* = 38), 38 and 32, respectively, completed the 6-month follow-up. Remission was maintained in 35/38 silymarin recipients and 21/32 controls among completers (OR = 6.11, 95% CI 1.53 to 24.45). A conservative intention-to-treat sensitivity analysis counted dropouts as not in remission and used the randomized denominators, giving an OR of 4.05 (95% CI 1.44 to 11.37; 35/42 versus 21/38). Both estimates favored silymarin, but their confidence intervals were wide (Figure 8). The trial was analyzed separately from induction studies because it addressed maintenance of remission, rather than induction in active disease.

### 3.11. Overall Efficacy Results for IBD Measures

The IBD-wide analyses provide an overall view of the randomized evidence, but they combine different polyphenol products and disease settings. Accordingly, the main text emphasizes clinical and endoscopic remission, whereas cross-product biomarker and quality-of-life analyses are presented as exploratory summaries.

#### 3.11.1. Clinical and Endoscopic Outcomes

For active native IBD, nine induction trials provided clinical-remission data from 229 polyphenol recipients and 176 controls. The random-effects estimate favored supplementation (OR = 6.13, 95% CI 3.31 to 11.38; *I*^2^ = 15%; Figure 9A). Trials of maintenance therapy and post-IPAA pouchitis were analyzed separately to account for their distinct clinical settings. Five studies reported endoscopic remission, with 113 intervention participants and 87 controls; pooling was restricted to remission outcomes, excluding endoscopic improvement and postoperative recurrence, and gave an OR of 9.33 (95% CI 1.93 to 45.04; *I*^2^ = 60.4%; Figure 9B). Although the endoscopic result favored supplementation, its magnitude remains uncertain because the confidence interval was wide and heterogeneity was moderate.

#### 3.11.2. Secondary and Exploratory IBD-Wide Outcomes

Three UC trials provided comparable post-treatment TNF-α data, and random-effects pooling showed no clear effect (SMD = −0.15, 95% CI −0.63 to 0.34; *I*^2^ = 55.6%). Martins AS et al. was considered separately because the study included a mixed UC/CD population and reported TNF-α on a log-transformed scale; its between-group ANCOVA was not statistically significant. MDA was available from three studies, with a random-effects SMD of −1.52 (95% CI −3.12 to 0.07; *p* = 0.061; *I*^2^ = 94.0%), indicating substantial heterogeneity and no statistically significant overall effect. Total IBDQ was available from two clinically different trials and is therefore presented as study-level estimates in the Supplement. Safety was evaluated separately by disease setting and event type rather than as a single overall IBD composite endpoint.

### 3.12. Efficacy of Dietary Polyphenols in Conventional UC

The UC evidence base was the largest in this review. The main text therefore focuses on disease activity, quality of life, systemic inflammation, and the principal clinical and endoscopic outcomes. Quantitative synthesis was undertaken when studies were sufficiently comparable in terms of clinical setting, outcome definition, and data structure. Outcomes for which meaningful quantitative synthesis was not considered appropriate are presented as study-level estimates or descriptive summaries in the Supplement.

#### 3.12.1. Disease Activity, Quality of Life, and Inflammation

SCCAI was reported as a post-intervention outcome in five RCTs involving 261 participants with UC (130 polyphenol, 131 control). Random-effects pooling showed lower SCCAI with supplementation (MD = −1.32, 95% CI −1.81 to −0.83; *I*^2^ = 0%; Figure 10A). Excluding Nikkhah-Bodaghi, which was at high risk for missing outcome data, gave a similar estimate (MD = −1.28, 95% CI −1.82 to −0.73; *I*^2^ = 0%). Three RCTs evaluated IBDQ-9 in 169 participants (83 intervention, 86 control); fixed-effect pooling showed higher IBDQ-9 scores (MD = 5.57, 95% CI 2.88 to 8.26; *I*^2^ = 0%; Figure 10B). Six studies reported post-treatment CRP/hs-CRP data (161 intervention, 127 control). In the three-arm Erol Dogan trial, the two active intervention arms were combined before comparison with the shared Mediterranean-diet control. Random-effects pooling showed no clear difference (SMD = 0.29, 95% CI −0.32 to 0.90; *p* = 0.357; *I*^2^ = 83.7%; Figure 10C). Excluding Amiri and Langmead, both at high risk for missing outcome data, did not reveal a treatment effect (SMD = −0.02, 95% CI −0.47 to 0.44).

#### 3.12.2. Clinical and Endoscopic Efficacy

Eight conventional UC trials reported clinical response data for 209 polyphenol recipients and 166 controls. Heterogeneity was low (*I*^2^ = 8%, *p* = 0.37); fixed-effect pooling showed higher odds of response with supplementation (OR = 4.90, 95% CI 3.04 to 7.89, *p* < 0.00001; Figure 11A). For remission during induction, eight UC trials contributed data from 209 intervention participants and 166 controls, giving a pooled OR of 5.98 (95% CI 3.08 to 11.59; *I*^2^ = 22.6%; Figure 11B). The result remained statistically significant and continued to favor supplementation after removing the Biedermann and Langmead trials, both judged at high risk of bias due to missing outcome data (OR = 7.31, 95% CI 3.46 to 15.43; *I*^2^ = 25.5%). Strict endoscopic remission was available from four UC studies (93 intervention and 77 control participants), with OR = 10.81 (95% CI 1.67 to 69.93; *I*^2^ = 69.4%; Figure 11C). Excluding Biedermann, which contributed per-protocol data and had high risk from missing outcome data, yielded OR = 24.62 (95% CI 7.76 to 78.15; *I*^2^ = 0%). The consistent direction but marked change in magnitude indicates that the endoscopic effect size is unstable.

#### 3.12.3. Secondary and Exploratory Outcomes

For ESR, three active-UC studies contributed data, and the pooled analysis showed no clear between-group difference (MD = −0.76 mm/h, 95% CI −5.07 to 3.54; *I*^2^ = 60.9%). Nikkhah-Bodaghi did not report ESR, whereas Rastegarpanah evaluated a maintenance population and was therefore considered separately. Fecal calprotectin was reported as mean ± SD in two UC trials. Ebrahimi Daryani reported 100.93 ± 167.11 versus 91.32 ± 158.36 μg/g (MD 9.61, 95% CI −96.75 to 115.97), whereas Biedermann reported 557 ± 756 versus 1040 ± 1179 μg/g (MD −483.00, 95% CI −1371.52 to 405.52). Given differences in intervention and analysis population, these estimates were presented separately rather than combined into a single summary effect. Ben-Horin reported fecal-calprotectin data in a form that could not be reliably converted to mean ± SD for this analysis. Mayo-related continuous outcomes were also summarized separately because the studies assessed different constructs, including post-treatment total Mayo score, change in endoscopic Mayo subscore, and change in partial Mayo score.

Pooling post-treatment TNF-α data from three conventional UC studies gave an SMD of −0.15 (95% CI −0.63 to 0.34; *I*^2^ = 55.6%; Supplementary Figure S5A), with no clear difference between groups. Other oxidative, hematologic, and symptom-related findings were reported individually when differences in treatment, clinical setting, outcome definition, or data structure made pooling difficult to interpret. Total antioxidant capacity was higher with resveratrol in Samsamikor (11.97 ± 1.61 vs. 9.41 ± 1.82; SMD = 1.47, 95% CI 0.88 to 2.06), but nearly identical between groups in Nikkhah-Bodaghi (2.16 ± 1.16 vs. 2.17 ± 1.17; SMD approximately 0). For MDA, the estimates favored supplementation in both studies: −3.25 in Samsamikor (95% CI −4.05 to −2.44) and −1.12 in Nikkhah-Bodaghi (95% CI −1.74 to −0.49). These SMDs were not pooled because the trials tested different polyphenol interventions under different measurement conditions. Amiri and Rastegarpanah each reported higher hemoglobin in the intervention arm. Marked baseline imbalance limited interpretation, as did the different clinical setting of Rastegarpanah’s maintenance trial. Biedermann’s symptomatic-remission outcome and Ben-Horin’s combined clinical-plus-objective co-primary response were also reported separately, since they measured different endpoints.

#### 3.12.4. Safety

Adverse-event reporting varied substantially across the UC trials, and event labels were not sufficiently uniform for a single patient-level composite analysis. Cutaneous or hypersensitivity-related events from Amiri, Rastegarpanah, and Sadeghi are therefore shown as study-level estimates without a pooled summary in the Supplement. Event-specific profiles are presented in Section 3.14. In the silymarin trial, several adverse-reaction categories were reported within the same participant cohort and cannot be summed as independent patient-level events. Overall, the small numbers and heterogeneous ascertainment preclude a precise comparative estimate of harm.

### 3.13. Efficacy Outcomes of Dietary Polyphenols for CD

#### 3.13.1. Clinical Remission

Evidence on remission induction in active CD came from Sugimoto et al. alone. CDAI-defined remission occurred in 8/20 participants receiving Theracurmin and 0/10 controls (study-level OR = 14.28, 95% CI 0.73 to 277.76). Bommelaer et al. examined recurrence prevention after ileocecal resection, so its postoperative results were not pooled with the induction trial because the clinical settings and estimands differed.

#### 3.13.2. Endoscopic Remission

For endoscopic outcomes, Sugimoto et al. reported induction of endoscopic remission in active CD (SES-CD ≤ 4): 3/20 versus 0/10 (study-level OR 4.20, 95% CI 0.20 to 89.61). Bommelaer et al. evaluated postoperative endoscopic recurrence (Rutgeerts ≥ i2a), which occurred in 21/31 curcumin recipients and 18/31 controls (OR 1.52, 95% CI 0.54 to 4.28). Because endoscopic remission in active CD and postoperative endoscopic recurrence are not interchangeable outcomes, no weighted CD summary was calculated; the two study-level estimates are presented separately in the Supplement.

#### 3.13.3. Adverse Events

Serious adverse-event data were available from the two CD trials and are presented as individual-study estimates in the Supplement. Because the trials differed in clinical setting and the event data were sparse, a combined estimate was not considered sufficiently informative. Bommelaer et al. reported five serious adverse events among 31 curcumin recipients and two among 31 controls (OR 2.79, 95% CI 0.50 to 15.62). Sugimoto et al. reported no serious adverse events in either the 20-participant intervention arm or the 10-participant control arm, so a relative effect was not estimable. These sparse data leave comparative serious-harm risk highly uncertain.

Bommelaer et al. reported event-specific serious adverse events including migraine, pregnancy, abdominal wall abscess, nausea, carcinoma of the uterine cervix, and abdominal pain (Figure 12). These categories arose from the same randomized population and are therefore displayed separately rather than summed or pooled. Event counts were low and confidence intervals were wide.

### 3.14. Event-Specific Adverse-Event Profiles

Individual adverse events were examined when trials reported them in sufficient detail. The aloe vera trial reported abdominal bloating, foot pain, sore throat, transient ankle swelling, acne, and worsening eczema as separate event categories. Counts were low, leaving the estimates imprecise (Figure 13a). In Sadeghi et al., skin allergy, dyspepsia, and heartburn all occurred in one participant receiving curcumin, with none reported in controls; these were not counted as independent patient-level events (Figure 13b). The silymarin trial reported headache, rash, diarrhea, abdominal pain, and nausea. These categories are displayed individually without a summed composite because more than one event may have occurred in the same person (Figure 13c). In Singla et al., no serious side effects occurred in either group. Worsening UC symptoms leading to treatment failure occurred in 5/23 versus 4/22 participants and are presented separately from the adverse-event categories (Figure 13d). These event-level analyses are descriptive and cannot establish equivalence or exclude uncommon harm.

## 4. Discussion

### 4.1. Clinical Efficacy and Mechanism of Action

The review covered seven polyphenol interventions in 19 randomized trials, reported in 21 publications and involving 1048 participants. That coverage was uneven, with most data coming from UC and a large share from curcumin studies. In UC, supplementation was favored in several pooled analyses of activity scores, selected inflammatory measures, and clinical or endoscopic outcomes. Evidence for CD consisted of two small trials conducted in different clinical settings. Conclusions are limited by differences in trials (formulation, dose, co-therapy, duration, event frequency). Figure 14 combines the clinical and biomarker findings with potential biological pathways to facilitate the interpretation of the observed effects, but does not mean that any one pathway has been established as a causal mediator.

#### 4.1.1. Levels of Treatment Response

The key finding is the spread of signals across patient-reported symptoms, systemic inflammation, mucosal inflammation, oxidative stress, and remission rather than any single biomarker. Symptom control is one of the short-term targets of Selecting Therapeutic Targets in Inflammatory Bowel Disease II (STRIDE-II) [46], while biomarker normalization is included in the intermediate phase, and steroid-free remission with endoscopic healing in the longer-term targets [46]. The outcomes in this review are therefore at different points of the treatment-response timeline.

Several outcome domains favored polyphenols, which is biologically coherent. Coherence is not causality. A conventional meta-analysis cannot determine whether modulation of NF-κB, Nrf2, the microbiota, or another pathway mediated remission, and the included trials rarely measured the intermediate steps required for such an analysis.

#### 4.1.2. Anti-Inflammatory and Antioxidant Networks

IBD is caused by genetic, environmental, microbial, epithelial, and immune factors. The preclinical literature indicates that some dietary polyphenols may target NF-κB-, MAPK-, NLRP3- and Nrf2/HO-1-related pathways with downstream effects on cytokine signaling, oxidative stress and epithelial injury [48,49,54]. These mechanistic observations provide biological context, but were not directly tested by most of the randomized trials included in this review.

The results of biomarker studies were inconsistent across various outcome domains. A clear effect on TNF-α (SMD = −0.15, 95% CI −0.63 to 0.34) or CRP/hs-CRP (SMD = 0.29, 95% CI −0.32 to 0.90) was not revealed by the pooled analyses. The triadic MDA evaluation revealed no statistically significant overall impact and exhibited considerable heterogeneity (SMD = −1.52, 95% CI −3.12 to 0.07; *I*^2^ = 94%). Study-level findings for fecal calprotectin, total antioxidant capacity and MDA differed by intervention and clinical setting, with some studies suggesting benefit and some little or no difference. Taken together, these findings cast doubt on the idea of a uniform class effect and instead point to the possibility that the antioxidant and anti-inflammatory effects of dietary polyphenols are likely to vary by compound, outcome measure, and clinical setting.

Oxidative injury and inflammation may interact in IBD, but a change in a single biomarker cannot be used to infer the pathway linking a supplement with a clinical outcome. Experimental work suggests plausible mechanisms including oxidative stress, mitochondrial function, epithelial injury, and antioxidant responses [48,49,54]. In this review, however, biomarker changes varied between compounds and were not consistently associated with symptom or endoscopic outcomes. Thus, the clinical data should be taken as evidence for efficacy rather than evidence that the effects are mediated by NF-κB, Nrf2 or mitochondria.

#### 4.1.3. Intestinal Barrier Repair

The epithelial barrier links luminal exposure with mucosal immune activation. Selected polyphenols have been shown to alter tight-junction proteins, MLCK-dependent myosin phosphorylation, epithelial apoptosis, and oxidative injury in experimental studies [49,54]. Few of the included RCTs measured permeability or junctional proteins directly. Accordingly, the barrier-related pathway in Figure 14 is used as a mechanistic link connecting the clinical and biomarker pattern with supportive biological evidence, rather than as proof of causal mediation. Limited human pharmacodynamic evidence of colonic AhR-CYP1A1 signaling is provided by CurQD, but the intervention includes both curcumin and QingDai, so findings cannot be attributed solely to curcumin [30].

In the CurQD trial, clinical and endoscopic improvement was accompanied by increased colonic CYP1A1 expression, which is consistent with aryl hydrocarbon receptor (AhR) engagement [30]. This is supportive human pharmacodynamic evidence, not proof that AhR activation mediated the clinical response, and it cannot be assigned specifically to curcumin because CurQD is a combination product.

#### 4.1.4. Microbiota-Mediated Metabolism

The clinical effects of dietary polyphenols could be related to microbial metabolism, as a number of phenolic compounds are modified by gut microorganisms and polyphenols and their metabolites may also affect the intestinal microbial environment [48,50,56,57]. In the current review, clinical and biomarker findings are used to provide part of the empirical basis for the mechanistic framework depicted in Figure 14 and microbiota-related pathways are used to link these observed effects to plausible biological mechanisms reported in the literature. The RCTs included did not directly verify microbiota-derived metabolites as mediators of remission, and therefore these pathways should be considered mechanistic links to help explain the meta-analytic findings rather than independently proven causal mechanisms. The CYP1A1 findings for CurQD are discussed separately above and represent a separate mechanistic signal, not direct evidence of microbial metabolism.

#### 4.1.5. Interpretation of Findings

A significant pooled estimate is not automatically clinically important, and neither is evidence of mechanism. Rare binary events can yield large ORs, especially when zero-event cells occur; broad CIs reveal the resulting imprecision. Differences in powder, nano-, self-emulsifying, enteric, or topical preparations also make a common biological effect unlikely. The collective pattern supports further study of polyphenols as add-on treatment. It does not justify applying one summary estimate to every compound, dose, and patient.

### 4.2. Interpretation of the Metabolism of Different Dietary Polyphenols

If the seven interventions were treated as equivalent, important differences would be obscured. They differ in first pass metabolism, chemical structure, colonic exposure, gastrointestinal (GI) stability, mixture complexity, and dependence on microbial transformation [13,48,49,50]. Using *p* values to generate a league table would not add much. The real question for clinical relevance is whether a formulation reaches target tissue at the point in disease progression where its proposed action may be of meaning.

Because these differences also determine how much confidence can be placed in each intervention, the following sections are organized by the strength of randomized evidence rather than by chemical class. Curcumin is discussed first, as it is supported by multiple RCTs and meta-analytic pooling. Resveratrol is then considered separately, because data come from more than one report but remain inconsistent. The remaining compounds—ginger, anthocyanins, aloe vera, *Achillea wilhelmsii*, and silymarin—are each supported by only a single small RCT; their findings are therefore presented as hypothesis-generating observations, not as established evidence of efficacy.

#### 4.2.1. Curcumin

Of the polyphenols reviewed here, the most extensive clinical data are for curcumin despite its poor water solubility. Effects on inflammatory signaling, oxidative stress responses and processes related to epithelial barrier have been described in experimental and translational studies [48,49]. In the randomized trials, clinical remission during induction was greater with curcumin, although endoscopic outcomes also generally favored curcumin-based supplementation. The endoscopic estimates were based on few events and were imprecise. Magnitude was influenced by study characteristics and risk of bias. Thus, curcumin is the most studied adjunctive polyphenol in the existing evidence base. However, the available data do not justify extrapolating its effects to all polyphenols, or using it as a substitute for the established standard therapy.

The efficacy of the exposure is likely more important than the labeled dose. Clinical trials have used conventional oral curcumin, nanomicelles, hydrophilic or self-microemulsifying formulations, combinations with piperine, enteric CurQD, rectal enemas and Theracurmin [19,30,36]. However, conventional low-dose preparations were not consistently effective, but several higher-dose, topical or bioavailability-enhanced regimens gave positive estimates [30,36,42,52]. The existing evidence supports a role as an adjunct rather than replacement for mesalazine, biologics or small molecules in mild-to-moderate UC [14,53].

Importantly, combination regimens should not be interpreted as evidence of curcumin monotherapy. CurQD contains QingDai, which is pharmacologically active and may independently engage aryl hydrocarbon receptor (AhR)-related pathways [30]. Similarly, curcumin administered with piperine [31,32] or probiotics [39] cannot be considered equivalent to curcumin alone, because piperine may enhance curcumin bioavailability, whereas probiotics may exert independent immunomodulatory effects. The He et al. trial also enrolled a clinically distinct post-IPAA pouchitis population rather than patients with conventional UC. Because pouchitis develops in a surgically constructed ileal pouch and is evaluated using pouch-specific clinical and endoscopic criteria, its pathophysiological and therapeutic context differs from that of conventional UC. We therefore retained this trial in the systematic review but excluded it from the conventional UC and overall IBD pooled analyses. Its findings should be interpreted specifically in the context of post-IPAA pouchitis and should not be generalized directly to patients with an intact colon. In patients with more severe UC, use of dietary supplements should not delay escalation of guideline-directed therapy.

#### 4.2.2. Resveratrol

In experimental studies, resveratrol has been associated with HO-1 upregulation, attenuated oxidative stress, suppression of inflammatory signaling, and epithelial barrier protection [48,54]. The human evidence was less straightforward. The two independent CRP/hs-CRP data sets, Samsami-kor SMD −0.45 (95% CI −1.02 to 0.12) and Erol Dogan SMD 0.37 (95% CI −0.33 to 1.07), went in opposite directions. Given the different contexts and directions of effect of the interventions, these results are interpreted at the study level. The data available do not show a systemic anti-inflammatory effect of resveratrol in UC.

In view of the current evidence, resveratrol is not recommended for standard therapy but as an investigational adjunct in mild-to-moderate UC. Future trials should combine plasma and stool metabolite profiling with validated clinical endpoints such as fecal calprotectin, endoscopic imaging, and patient-reported measures to lend support to more definitive conclusions.

#### 4.2.3. Other Polyphenols: Single Small Trials as Hypothesis-Generating Evidence

The other compounds are ginger, anthocyanins, aloe vera, *Achillea wilhelmsii*, and silymarin, each supported by only one small RCT. Consequently, their discoveries are presented as hypothesis-generating observations rather than as established evidence of efficacy. The lack of endoscopic or histological confirmation, short duration, small sample size, single-center or prematurely terminated design, internal data inconsistency, and lack of support from meta-analytic pooling are the limitations of each study.

(1) Ginger. The major phenolic compounds of ginger, the gingerols and shogaols, have shown antioxidant and anti-inflammatory activities in preclinical studies, mainly via the inhibition of ROS generation and the protection of membrane lipids against oxidative damage. Yet, only one small UC clinical trial has evaluated ginger (*n* = 46 completers; 22 ginger, 24 placebo; 12 weeks). In that trial, MDA and clinical disease scores improved compared to placebo, but overall TAC did not change meaningfully [41]. These results are consistent with reduced lipid peroxidation in this small single trial, but do not establish a causal relationship of reduced MDA with symptom relief, nor do they demonstrate endoscopic or histologic benefit. Other limitations are the small sample, short duration, single center design, lack of meta-analytic replication and variability of crude ginger powder according to farming and processing conditions. This finding should therefore be considered as only hypothesis-generating. Standardized, well-characterized formulations and multicenter designs will be required for robust replication.
(2) Anthocyanins. The only randomized data on anthocyanins in active UC is a phase IIa trial that was prematurely terminated due to COVID-19. Thirty-four patients were randomized but the per-protocol analysis included 18 ACRE and eight placebo patients. ACRE led to improvement in fecal calprotectin (*p* = 0.035) but no significant improvement in clinical and endoscopic endpoints and a large placebo effect was noted [35]. These findings may be due to recruitment disruption, substantial underpowering, short-term physiologic fluctuation, or statistical noise. This one, a per-protocol analysis that was aborted, is only a tentative hypothesis, not conclusive proof of efficacy. Further larger prospective trials are warranted to elucidate the potential of calprotectin trajectories as a marker for preceding clinical symptom resolution and true endoscopic mucosal healing.
(3) Aloe vera gel. Aloe vera gel has local mucosal protective and wound healing effects in preclinical studies, attributed to the polysaccharides and phenolic constituents. However, only one UC trial was included, a 4-week double-blind RCT of 44 evaluable patients (aloe 30, placebo 14). It showed a higher clinical response rate with aloe vera but clinical remission and clinical improvement did not reach significance and sigmoidoscopic and histological outcomes showed no clear benefit [40]. A 4-week intervention might be sufficient to relieve symptoms but not to achieve mucosal healing; measurement bias and chance might also have played a role. Also, the amount of polysaccharide, phenolic and anthraquinone may vary with extraction and purification, and anthraquinones may cause diarrhea and interfere with results. This single small short-term trial therefore provides evidence of a possible symptomatic effect, but not evidence of endoscopic or histological remission. It is hypothesis-generating. Currently there is no evidence for efficacy of aloe vera gel as a single-agent therapy in inducing mucosal remission. Future work should define chemical composition and strict upper limits for laxative constituents.
(4) *Achillea wilhelmsii* and silymarin. *Achillea wilhelmsii* contains flavonoids and phenolic compounds with in vitro antioxidant and antispasmodic activity. However, the only included clinical trial found no statistically significant between-group difference in improvement from baseline [43]. As a single small trial, it is hypothesis-generating rather than definitive evidence for or against the preparation. Silymarin is rich in silybin and has shown antioxidant and membrane-stabilizing effects in preclinical studies. Only one randomized trial evaluated silymarin for maintenance of UC remission [44]. Among completers, remission was maintained in 35/38 participants receiving silymarin and 21/32 receiving placebo (OR 6.11, 95% CI 1.53 to 24.45). A conservative intention-to-treat sensitivity analysis using the randomized denominators and counting dropouts as non-remission yielded 35/42 versus 21/38 (OR 4.05, 95% CI 1.44 to 11.37). Because this trial addressed maintenance rather than induction of remission, it was analyzed separately from active-disease induction studies. Independent maintenance trials using prospectively specified intention-to-treat analyses and objective clinical and endoscopic outcomes are needed.

### 4.3. Disease Heterogeneity and Clinical Characterization

Most pooled IBD estimates in this review are, in effect, UC estimates; they should not be carried over to CD. In UC, inflammation is continuous, superficial, and colonic, so an orally retained or rectally delivered compound may contact the affected mucosa. CD is discontinuous and transmural, may occur anywhere in the gastrointestinal tract, and includes stricturing, fistulizing, and postoperative phenotypes [55,58]. A mainly luminal antioxidant action may be inadequate for those deeper processes. Separate claims for the two diseases are therefore necessary [46].

Dietary polyphenols should be explored only as adjuncts to established therapy for mild-to-moderate UC and not as replacements for evidence-based treatment. Treatment decisions should be based on disease severity and risk when disease activity persists or escalates; supplementation should not delay corticosteroids, biologics, small molecules or other indicated therapy [53]. Other considerations are product quality, drug interactions, organ function, risk of bleeding, and cost.

The CD literature comprises two randomized trials with clinical differences. Sugimoto et al. studied induction outcomes in active mild-to-moderate CD patients, while Bommelaer et al. examined prevention of postoperative recurrence [33,37]. These trials were not combined. Sugimoto reported a potential clinical-remission signal but with a zero-event control arm and a very wide confidence interval; its endoscopic-remission estimate was similarly imprecise. Bommelaer did not assess induction of remission and did not show a clear reduction in postoperative endoscopic recurrence. Current CD management, in contrast, is focused on the phenotype, location of lesions, complications, and risk of progression [55,58]. Polyphenols are still under investigation in CD.

Supplement exposure cannot be completely separated from the remainder of the diet. The ESPEN clinical-nutrition guidelines emphasize the nutritional assessment, diagnosis of malnutrition and integration of dietary management with medical treatment [59]. Instead of treating the compound as an isolated drug [50,56], precision nutrition would put a supplement in that context—diet, medication, phenotype and microbial capacity of the patient to metabolize it. Reported diet-microbiota associations provide biological context, but do not establish supplement-specific efficacy [57].

### 4.4. From Mechanistic Rationality to Precise Clinical Applications

What is largely lacking is a link between administered dose and clinical outcome. NF-kB, Nrf2, NLRP3, junctional proteins, and the microbiota have been shown to be altered in cell and animal studies. The majority of clinical trials record a dose and then an endpoint. They infrequently measure plasma concentration, fecal metabolites, mucosal exposure or target engagement. A negative result can thus mean that the mechanism is inactive or simply that too little active compound has reached the relevant tissue. Existing studies are unable to tease out between these possibilities.

Interpretation of dose requires reproducible formulation. Reports are required on source of raw material, batch-to-batch variation, storage conditions, dissolution, purity, stability and active content. Only then is there use in comparison of enteric, topical, nano-, self-emulsifying, lipid or conventional delivery [31,49]. Additionally, multi-herb preparations demand a chemical fingerprint and monitoring for potentially harmful constituents. Without this information, the basis for dose–response analysis is uncertain.

A useful pharmacodynamic design would include sampling beyond CRP and ESR. Plasma or fecal metabolites and redox measures may accompany fecal calprotectin, centrally read endoscopy, histology, mucosal transcriptomics, junctional proteins, AhR–CYP1A1, or microbial function based on the stated hypothesis [30,50,56,57]. Timing is everything. Repeated measures can establish whether exposure predates biomarker and clinical change and can support a prespecified mediation analysis prior to unblinding.

It is unlikely that an average treatment effect will cover every patient. Measured metabolite exposure, lesion location, concomitant therapy, disease subtype, microbial features, diet quality, and baseline activity are all likely effect modifiers that can be specified in advance. Reporting a significant subgroup *p* value is not the same as showing interaction. The interaction term itself has to be tested. For a small early phase program a Bayesian hierarchical model could borrow some information across formulations but also present the probability of benefit, rather than reduce interpretation to *p* < 0.05.

Induction and maintenance should not have one protocol. Clinical and endoscopic response, change in fecal-calprotectin and steroid-free remission are required for a trial in active UC; maintenance is better judged by time to relapse, endoscopic recurrence, escalation, hospitalization and cumulative corticosteroid exposure. CD also needs differentiation of active luminal, perianal and postoperative settings. Pooling such cohorts might obscure rather than reveal a real effect.

Power calculations should begin with a clinically important difference rather than the smallest sample possible. Minimum important change should be found for continuous outcomes, a priori. For binary endpoints the OR should be accompanied by the number needed to treat, relative risk and absolute risk difference. Sparse events may require methods that are exact, mixed or Bayesian, as well as a sensitivity analysis for zero cells. The protocol also calls for hierarchy or another explicit approach to multiplicity when many related outcomes are tested.

Another problem is how it is used in practice. Commercial supplements vary greatly in composition, quality, and dose. A practical pathway would be dietitian review with a standardized product, early objective assessment of response and clear rules for discontinuation of supplementation or up-titration of conventional therapy. If polyphenols have a role it is as evidence-based adjunct to individualized nutritional management. They do not offer a natural alternative to disease control.

### 4.5. Safety and Tolerability

Adverse-event reporting was incomplete, with few events reported. There was no consistent pattern of increased harm across trials, although in a few individual trials the number of events was higher in participants receiving polyphenols, notably in the silymarin trial. Event categories were often non-uniform and multiple categories within one trial could appear in the same participant, making summation into a patient-level composite misleading. No consistent pattern of recurrent organ toxicity or serious infection was seen, but the trials were too small and too short to exclude infrequent adverse effects. The correct inference is that there is a paucity of evidence of short-term tolerability, not that safety has been demonstrated.

### 4.6. Clinical Implications

Polyphenols should be administered as an adjunct to standard therapy, not as a replacement. The phrase used for curcumin—the largest randomized clinical evidence base of the polyphenols assessed in this review, mainly in UC—describes the quantity of randomized evidence and is not a formal certainty grade. There is still little proof for resveratrol and the other products. In practice, the exact formulation and dose should be noted and efficacy should be evaluated by means of objective measures of inflammation and not just symptoms. Uncontrolled bleeding, persistent elevated inflammatory markers and moderate-to-severe disease require timely escalation of guideline-directed therapy. Adjustment of a polyphenol supplement is not an acceptable alternative to indicated medical management.

### 4.7. Strengths and Limitations of the Study

This review synthesizes evidence across seven polyphenol or polyphenol-rich interventions while retaining outcomes ranging from symptoms and oxidative stress to endoscopy and adverse events. The available data also allowed product-specific findings to be distinguished from disease-specific findings in UC and CD. Another strength is interpretative: Table 2 separates clinical observations from mechanistic evidence derived mainly from experimental studies.

The conclusions are materially affected by several limitations. Most participants had UC and much of the evidence for the interventions was related to curcumin. The trials were generally small and short with significant heterogeneity in endpoint definitions, routes of administration, formulations, doses, background therapies and follow-up. For many outcomes there were not enough studies to reliably assess publication bias. Reports of hospitalization, surgery, steroid withdrawal and long-term relapse were rare. Some trials were terminated early, used per-protocol or completer analyses, or had inconsistent statistical reporting. Methodological concerns were identified in a substantial proportion of trials by RoB 2, with missing outcome data being a major contributor to high-risk judgements; trial-level judgements and their missing data basis are provided in Supplementary Table S2. Sensitivity analyses excluding Domain 3 high-risk results are summarized in Supplementary Table S3. These analyses suggested that the primary clinical-remission results were directionally robust, but the magnitude of the endoscopic-remission effect was less stable. Formal GRADE ratings were not prespecified in the protocol and therefore were not introduced post hoc. Instead, outcome-specific RoB 2 judgements, clinical and statistical heterogeneity, precision of the confidence intervals, indirectness, and sensitivity analyses addressed the limitations of certainty. This approach should be taken into consideration when comparing the present review with reviews that provide formal GRADE categories.

An additional limitation is overlap within the evidence base. Although 21 reports were included, they represented 19 independent randomized trials, because some publications were companion reports from the same underlying study. These reports were linked at the trial level and were not counted as separate independent trials within any individual pooled analysis. In addition, because the review examined the evidence from different analytical perspectives, the same eligible trial could contribute to a product-specific analysis, an overall IBD analysis, and a disease-specific analysis when it reported the relevant outcome. These analyses therefore represent different views of a substantially overlapping evidence base and should not be interpreted as independent replications of the same treatment effect.

Sparse events and zero-event cells also limit the interpretation of several dichotomous outcomes. Some remission analyses contained few events, producing large odds ratios with wide confidence intervals. In such settings, the pooled estimate can be strongly influenced by only a small number of events and, where continuity corrections are required, by the handling of zero-event cells. Trials with no events in either arm provide no information on the relative treatment effect. Accordingly, these estimates are more informative about the possible direction of effect than about its precise magnitude. This limitation is particularly relevant to clinical and endoscopic remission outcomes, where sparse events and non-uniform endpoint definitions further increase uncertainty.

UC and CD also differ biologically and clinically, which limits interpretation of pooled estimates across disease phenotypes. Finally, Figure 14 is based largely on the preclinical and other supportive mechanistic literature and should not be interpreted as a mechanistic result of this meta-analysis. Taken together, the small evidence base, overlapping analytical frameworks, sparse events, and methodological heterogeneity reduce confidence in the precision and generalizability of several pooled estimates.

### 4.8. Future Research Priorities

The next generation of trials needs more than a larger sample. Multicenter, double-blind designs should use batch-controlled formulations and adequate power. UC and CD require separate studies, as do induction and maintenance, with consistent clinical, endoscopic, and histological definitions. Follow-up must be long enough to record steroid-free remission, hospitalization, surgery, and escalation. Within the same protocol, pharmacokinetics should be linked to mucosal pharmacodynamics and microbial metabolism. Subgroup and mediation analyses belong in the registration, and interaction and safety reporting must be complete. Multi-omics prediction will be valuable only when responder definitions are interpretable and reproduce outside the development data set.

## 5. Conclusions

Randomized trials suggest that dietary polyphenols, especially curcumin-based formulations, may provide adjunctive benefit for selected clinical outcomes in UC. Although there was a general preference for supplementation, clinical remission during induction and SCCAI generally favored supplementation, whereas pooled analyses of CRP/hs-CRP and TNF-α did not demonstrate clear reductions. Endoscopic remission was also in favor of supplementation, but the estimate was still imprecise and subject to sparse events, heterogeneity between studies and risk of bias. Evidence in CD is still scarce and clinically heterogeneous, precluding a reliable overall estimate. Dietary polyphenols should thus be regarded as experimental adjunctive treatments, not as substitutes for established IBD treatment.

Future trials should use chemically characterized formulations and adequate sample sizes. They should clearly distinguish between induction and maintenance treatment and use standardized definitions for outcomes. Clinical response and remission should be assessed with central review of endoscopy, fecal calprotectin, steroid-free remission, relapse or treatment escalation and systematic assessment of adverse events. Analyses should be by intention to treat. An effort should be made to minimize missing outcome data so that estimates of treatment effect are more reliable and generalisable.

## Figures and Tables

**Figure 1:**
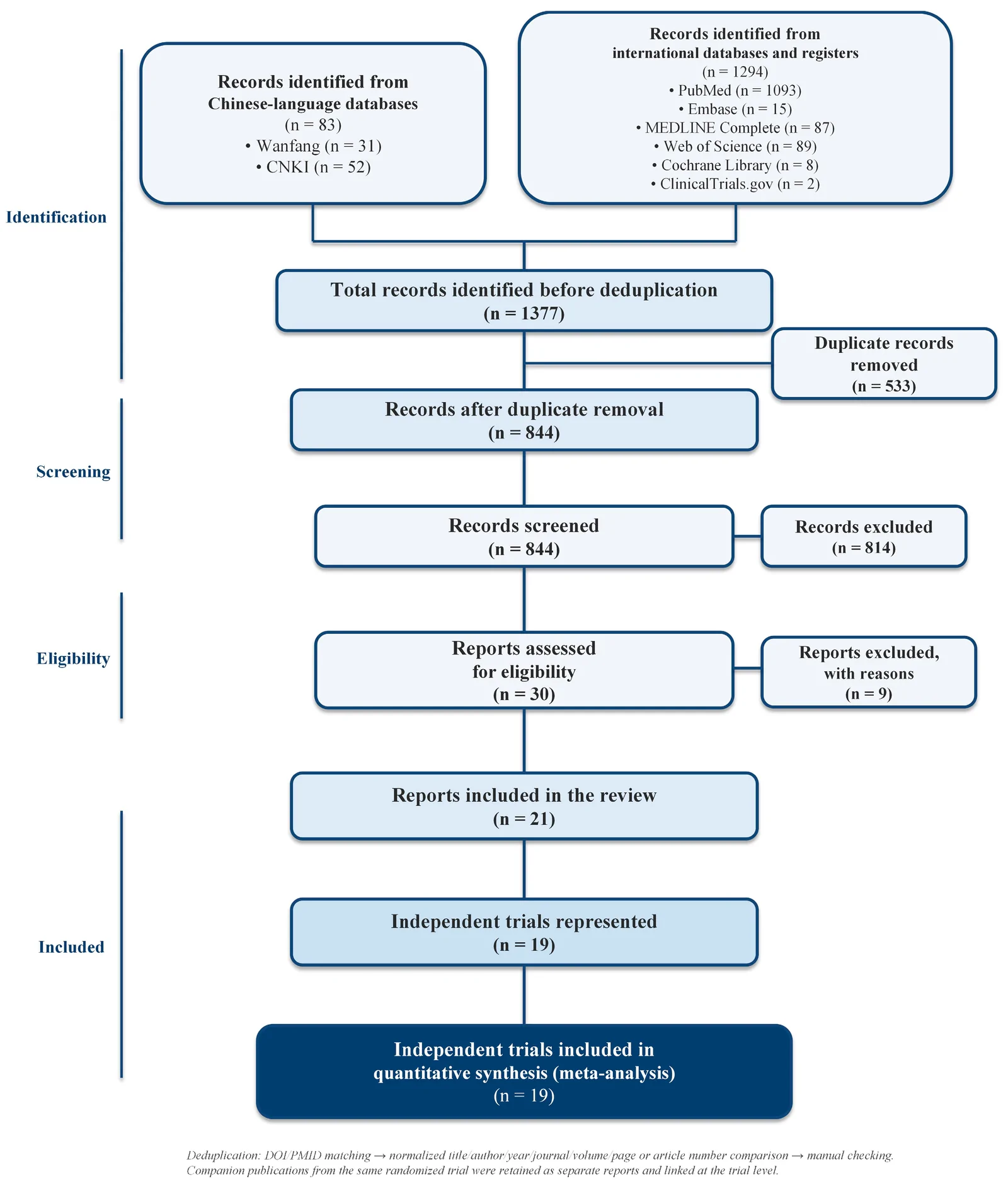
Study selection flowchart.

**Figure 2:**
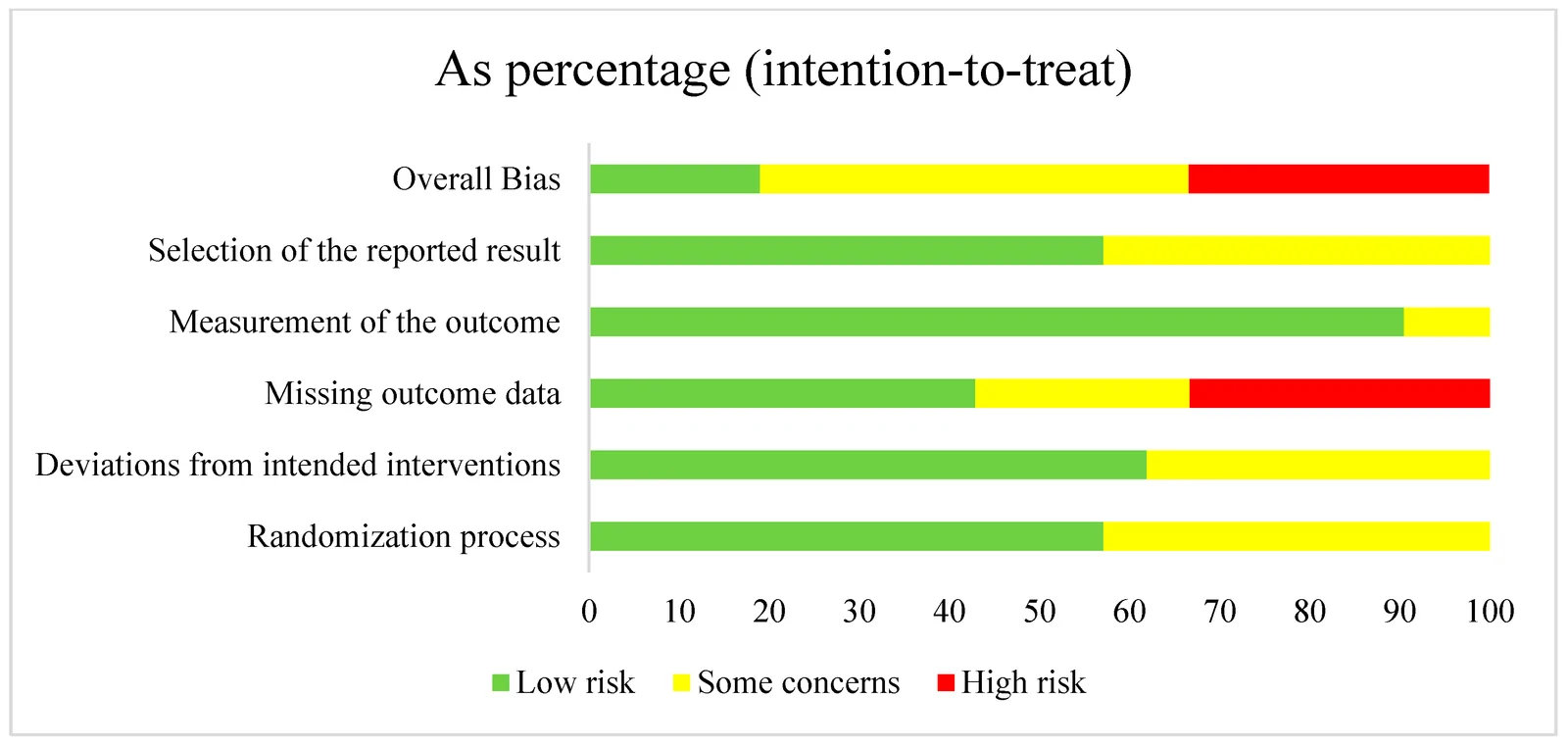
Summary of risk-of-bias judgments for evaluated trial results using the Cochrane RoB 2 tool.

**Figure 3:**
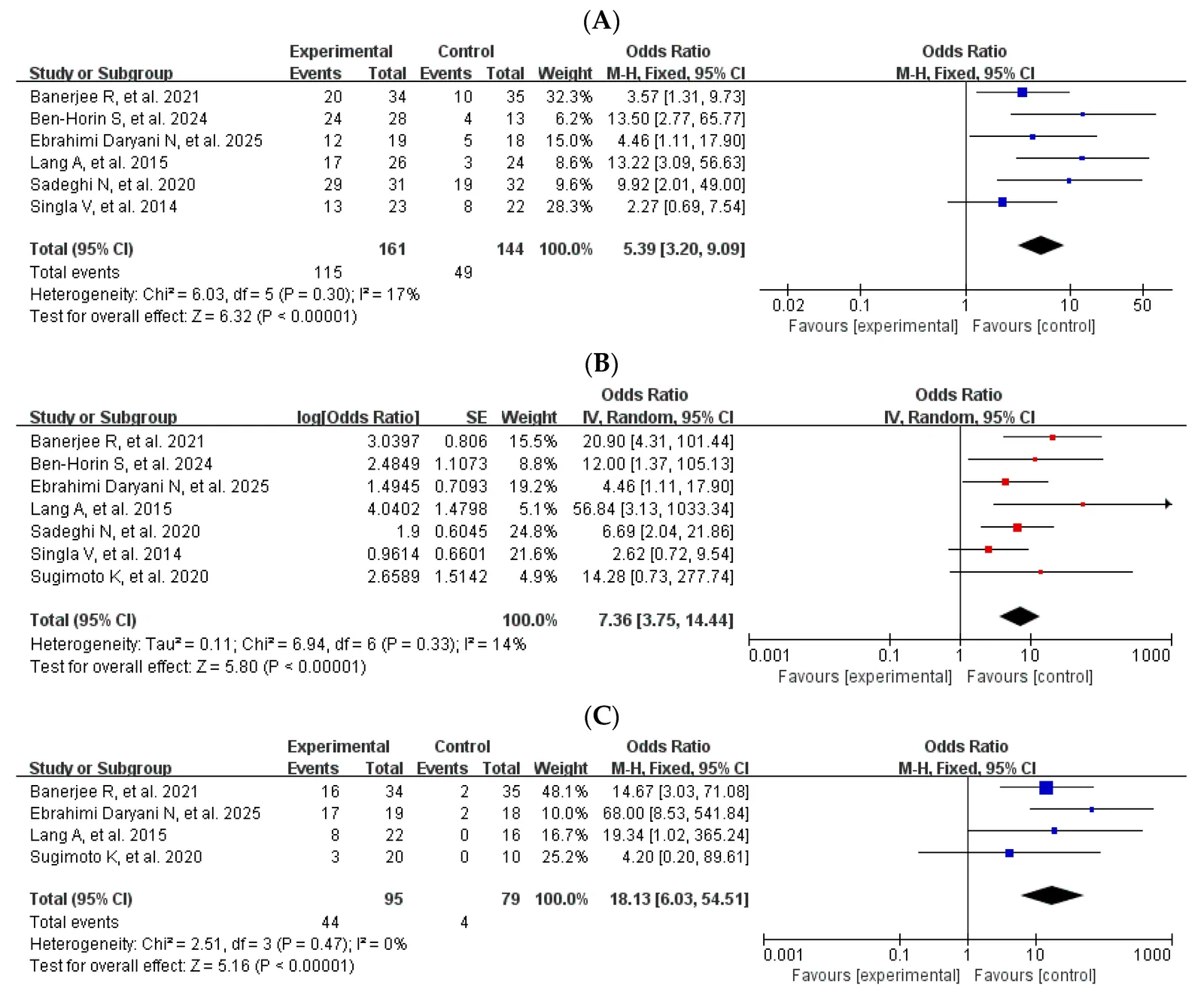
Key clinical outcomes of curcumin-based supplementation. (**A**) Clinical response. (**B**) Induction of clinical remission in active native IBD. (**C**) Endoscopic remission in active native IBD [20,30,34,36,37,42,45]. Colored squares represent individual study estimates, horizontal lines indicate 95% CIs, black diamonds denote pooled effects, and the vertical line at OR = 1 indicates no effect.

**Figure 4:**
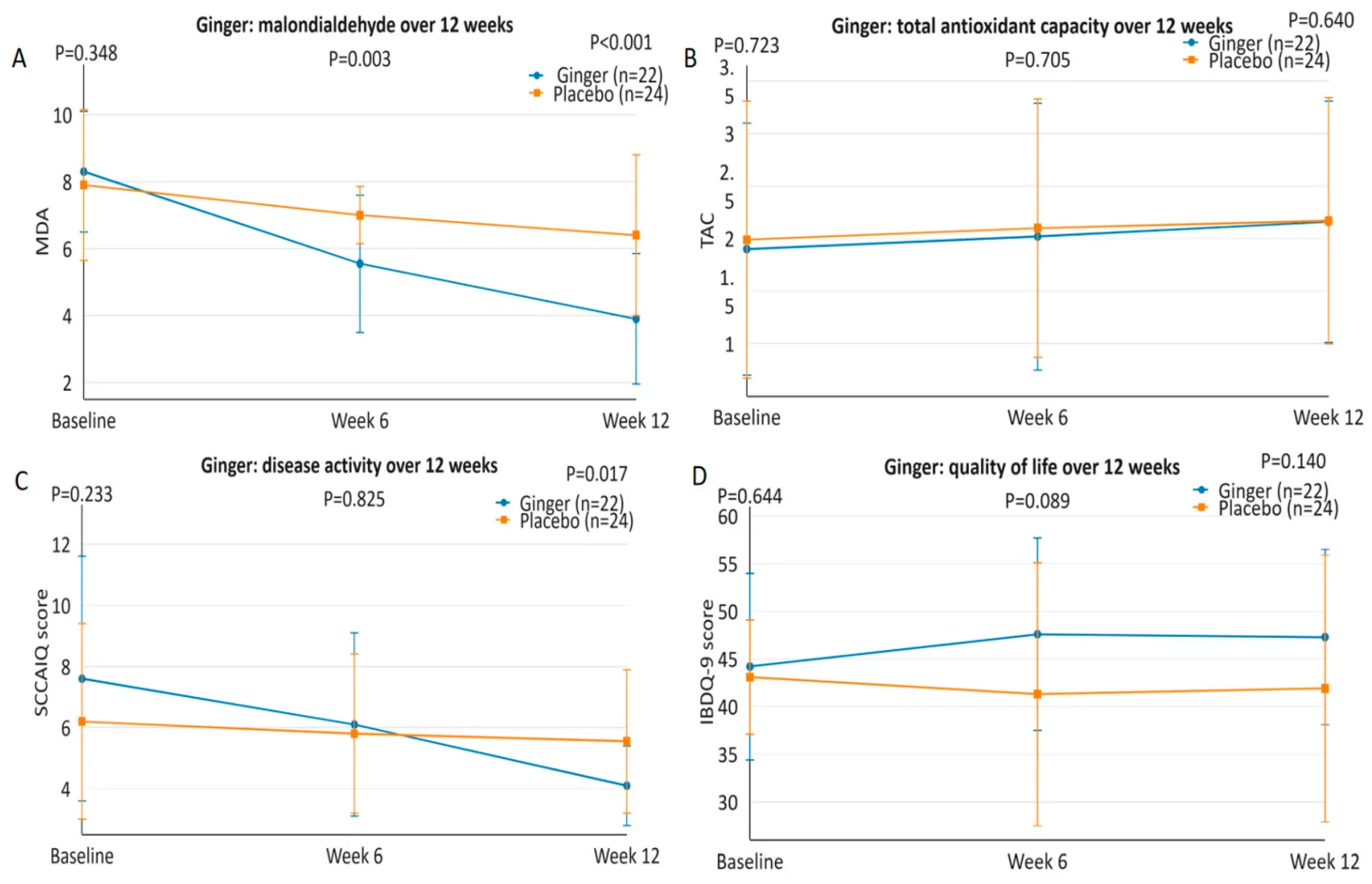
Oxidative stress, disease activity, and quality-of-life outcomes in the ginger trial. Note: Panels show MDA (**A**), TAC (**B**), SCCAI (**C**), and IBDQ-9 (**D**) at the stated assessment times. Values are mean ± SD; figure *p* values are between-group comparisons at each time point.

**Figure 5:**
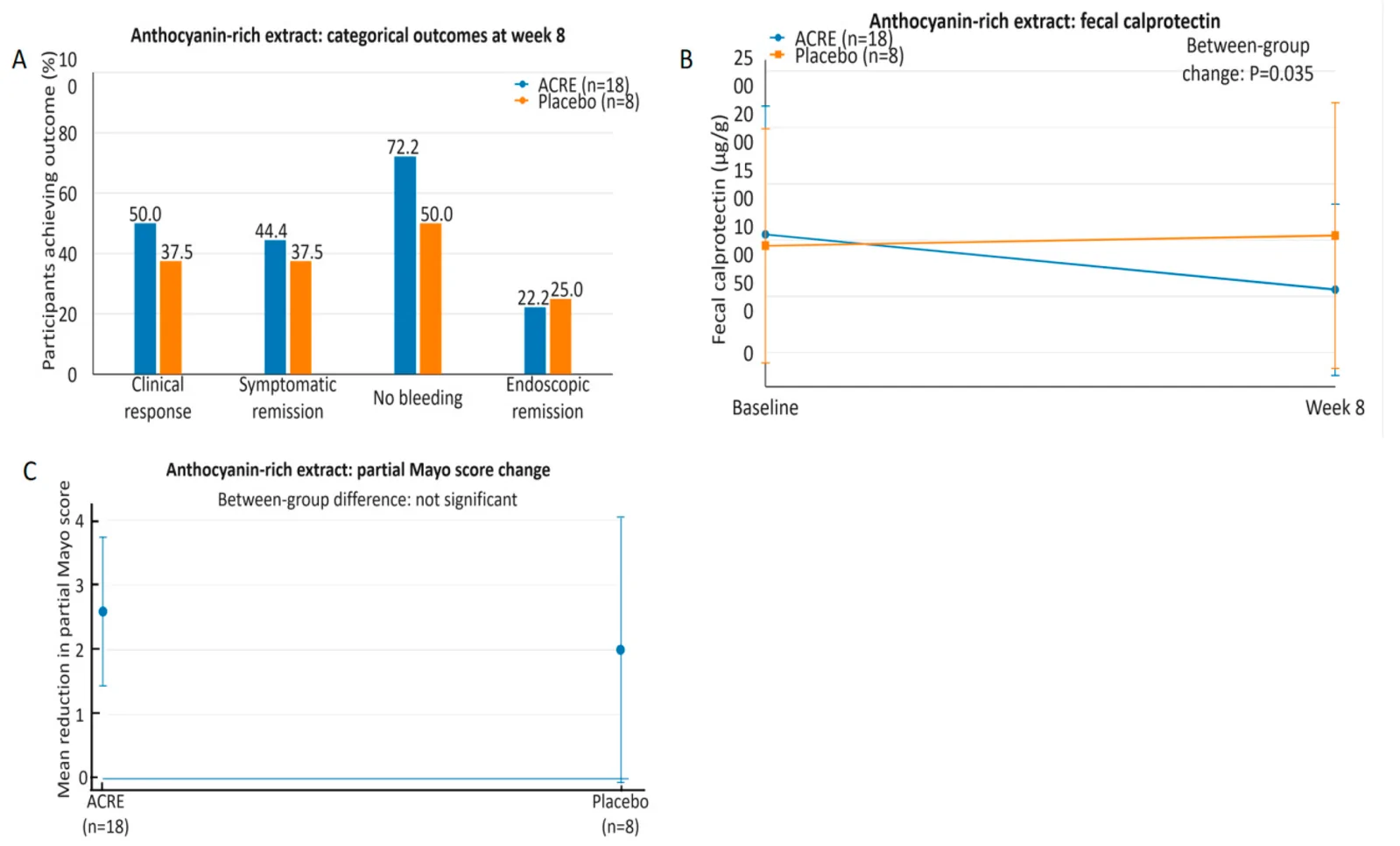
Clinical and biochemical findings for anthocyanin-rich extract in UC. Note: At week 8, panel (**A**) reports absence of rectal bleeding, symptom remission, endoscopic remission, and clinical response. Panel (**B**) shows the change in fecal calprotectin as mean ± SD, and panel (**C**) shows the mean decrease in partial Mayo score with 90% CIs. Categorical plots were restricted to outcomes with concordant event counts and percentages.

**Figure 6:**
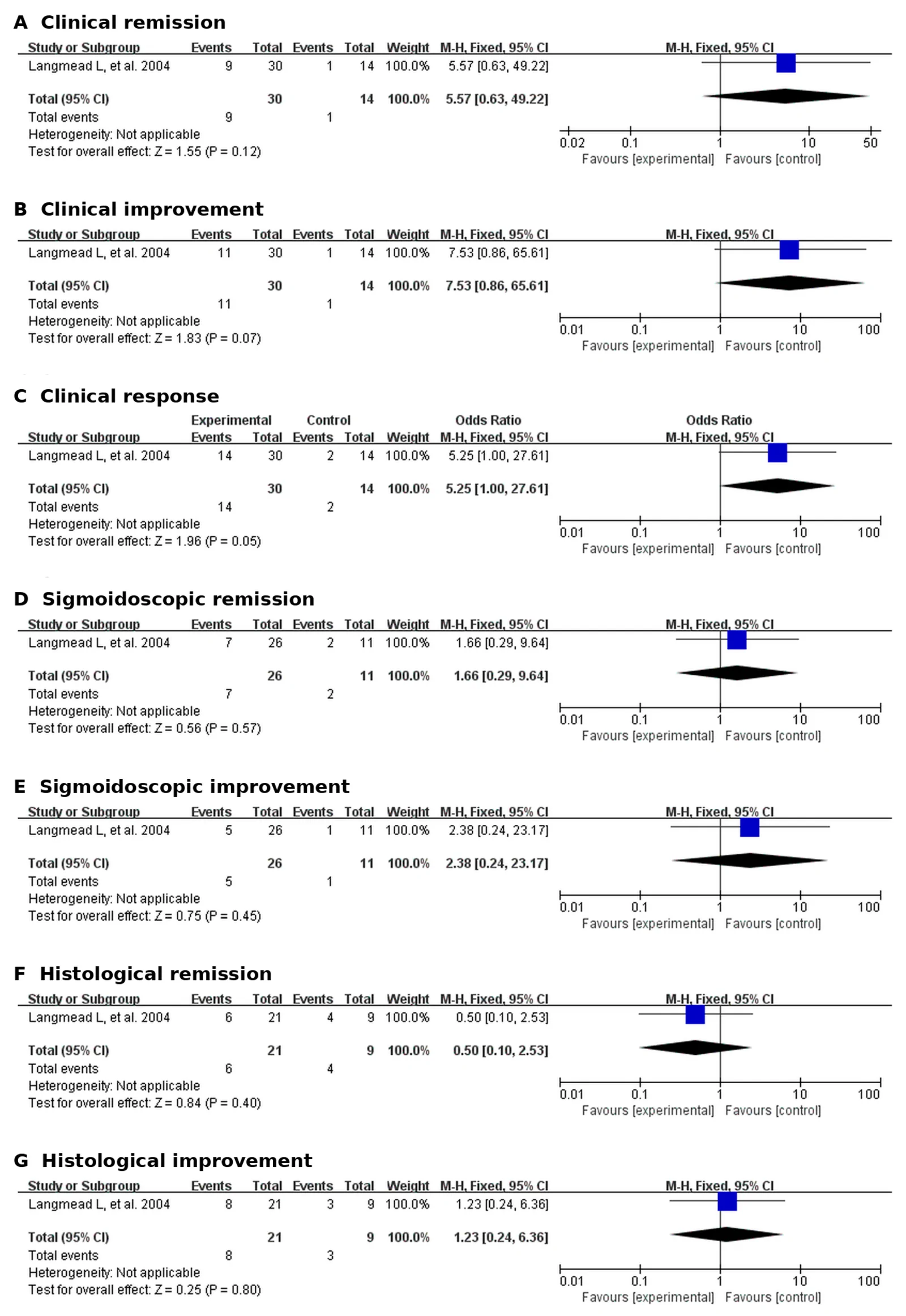
Study-level efficacy outcomes for oral aloe vera gel in UC: (**A**) clinical remission; (**B**) clinical improvement; (**C**) clinical response; (**D**) sigmoidoscopic remission; (**E**) sigmoidoscopic improvement; (**F**) histological remission; and (**G**) histological improvement. Odds ratios and 95% CIs are shown for the single included randomized trial; the no-effect value is 1.0 [40]. Blue squares represent study-specific estimates, horizontal lines indicate 95% CIs, the vertical line at OR = 1 indicates no effect.

**Figure 7:**
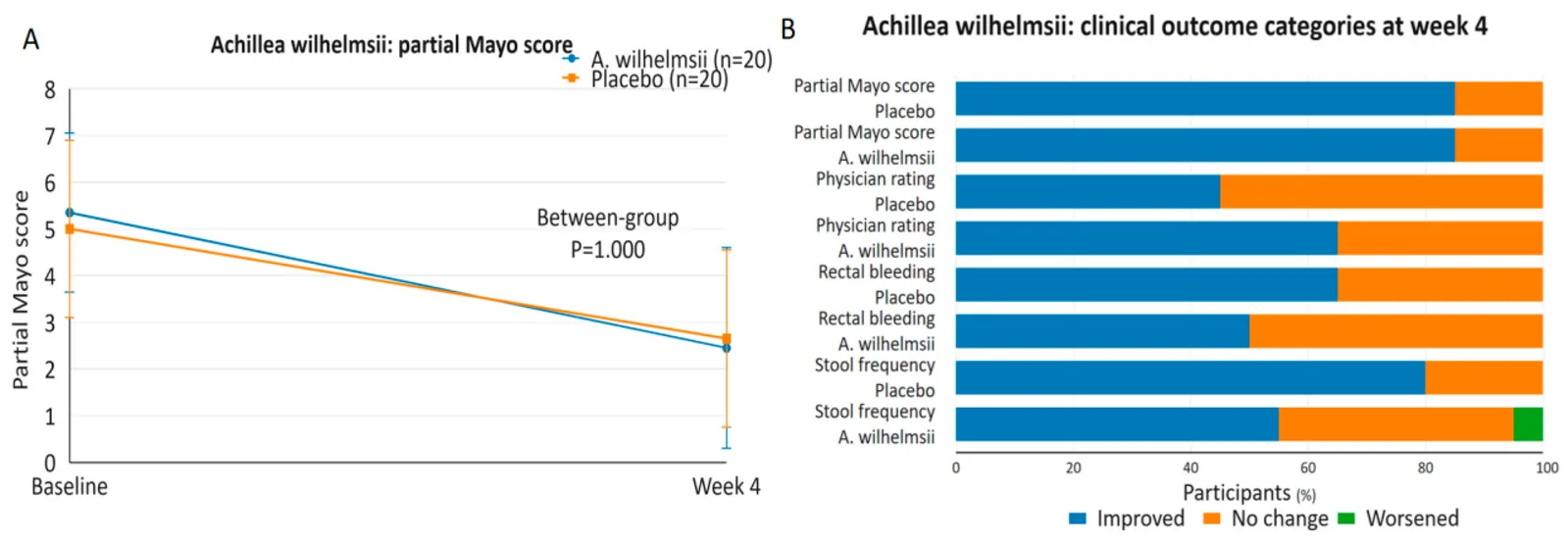
Four-week outcomes for *Achillea wilhelmsii* in UC. Note: Partial Mayo score at enrollment and week 4 is reported as mean ± SD in panel (**A**); panel (**B**) covers the changes in stool frequency, rectal bleeding, physician assessment, and partial Mayo score, with no significant difference between the study arms for any measure.

**Figure 8:**
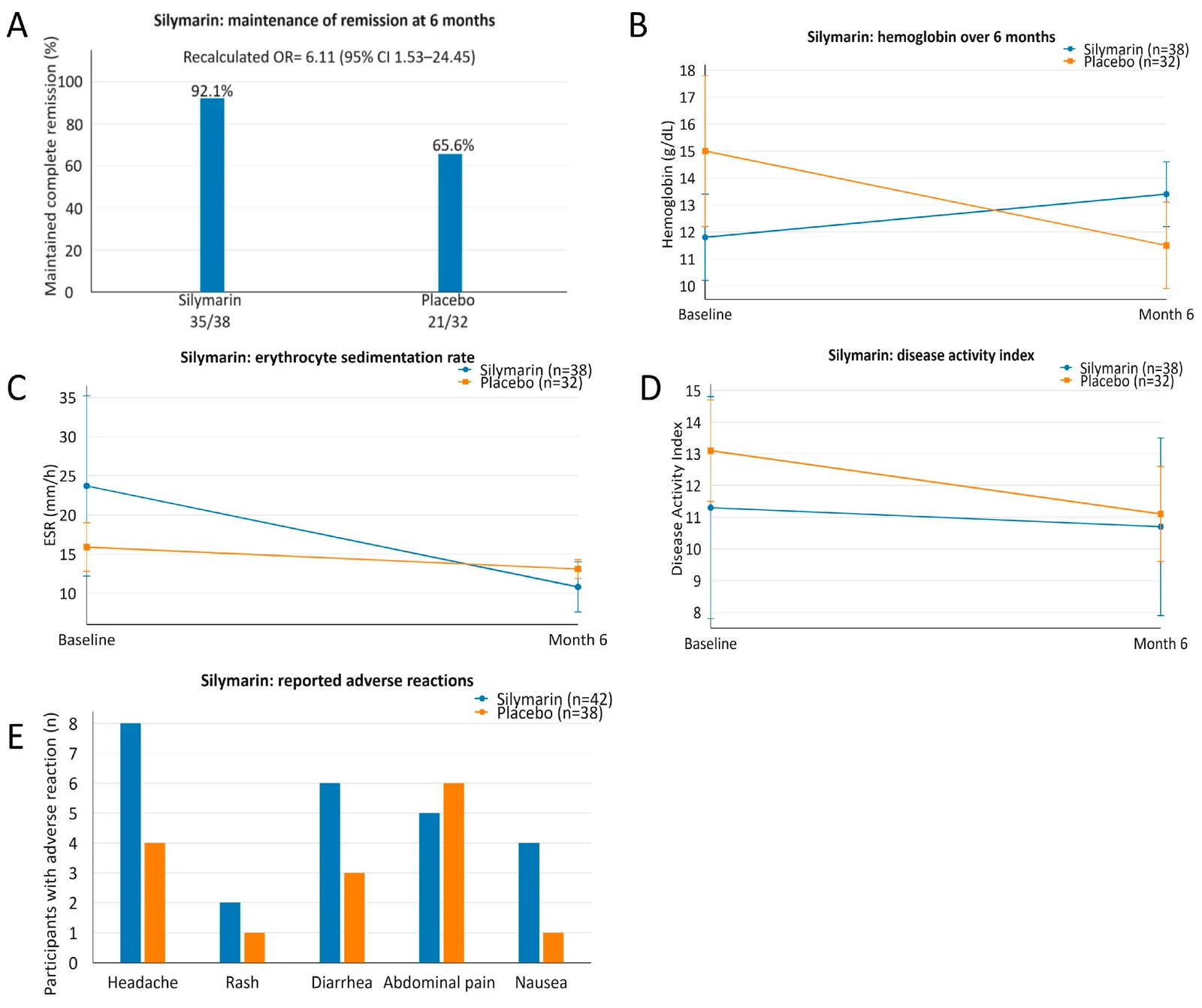
Silymarin trial: maintenance of remission, laboratory findings, disease activity, and event-specific adverse reactions. Note: Panel (**A**) shows the published completer analysis for maintenance of remission at 6 months (35/38 vs. 21/32; OR 6.11, 95% CI 1.53 to 24.45). Because attrition occurred after randomization, an intention-to-treat sensitivity analysis was also performed by treating dropouts as non-remission (35/42 vs. 21/38; OR 4.05, 95% CI 1.44 to 11.37) and is reported in the text and Supplementary Materials. Panels (**B**–**D**) show descriptive changes in hemoglobin, ESR, and disease activity index among study completers. Panel (**E**) presents event-specific adverse reactions separately rather than as a pooled composite endpoint.

**Figure 9:**
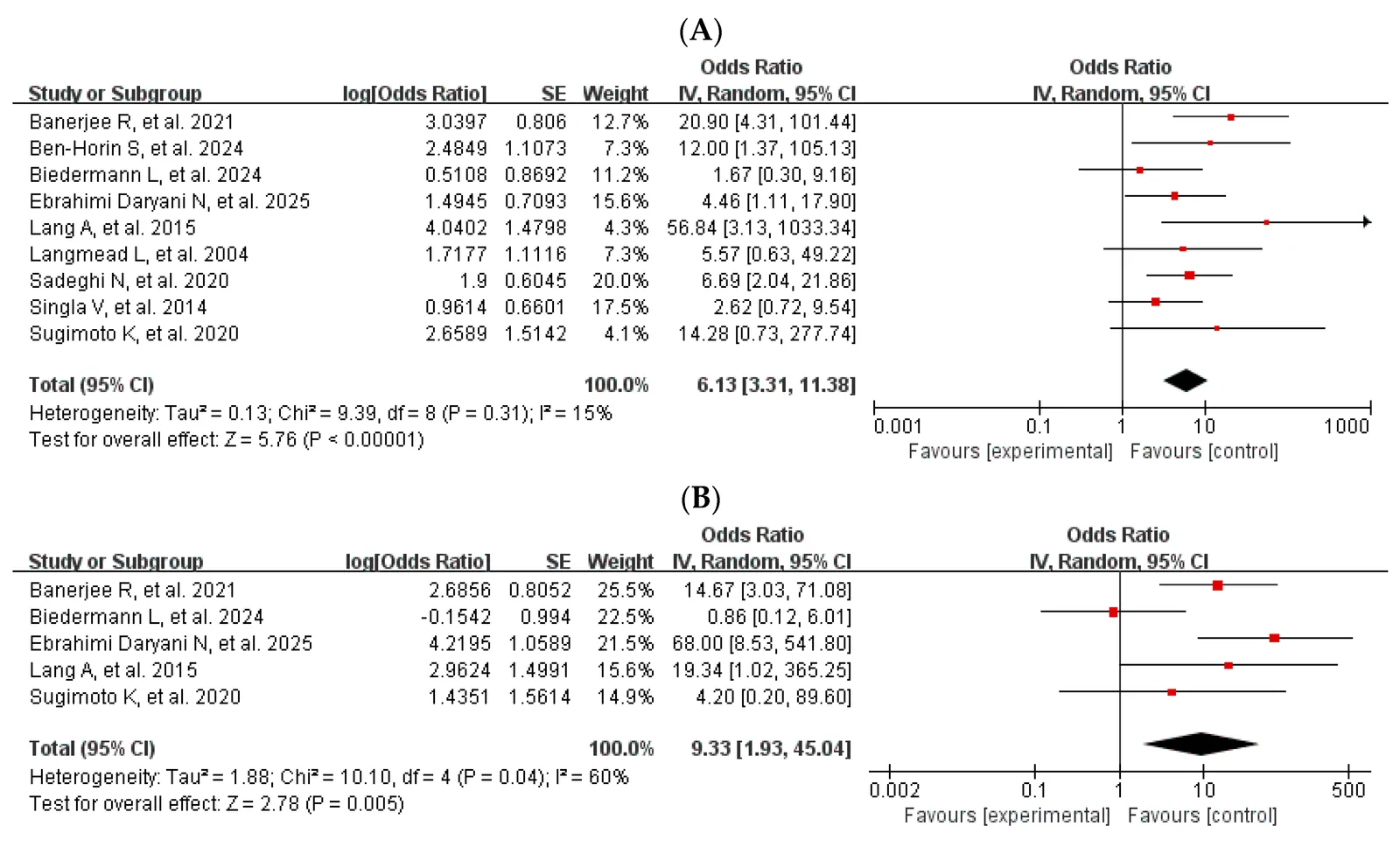
Key induction outcomes of dietary polyphenols in active native IBD. (**A**) Clinical remission. (**B**) Endoscopic remission. Maintenance-of-remission, postoperative-recurrence, post-IPAA pouchitis, and endoscopic-improvement-only outcomes were excluded from these pooled analyses [20,30,34,35,36,37,40,42,45]. Colored squares represent individual study estimates, horizontal lines indicate 95% CIs, black diamonds denote pooled effects, and the vertical line at OR = 1 indicates no effect.

**Figure 10:**
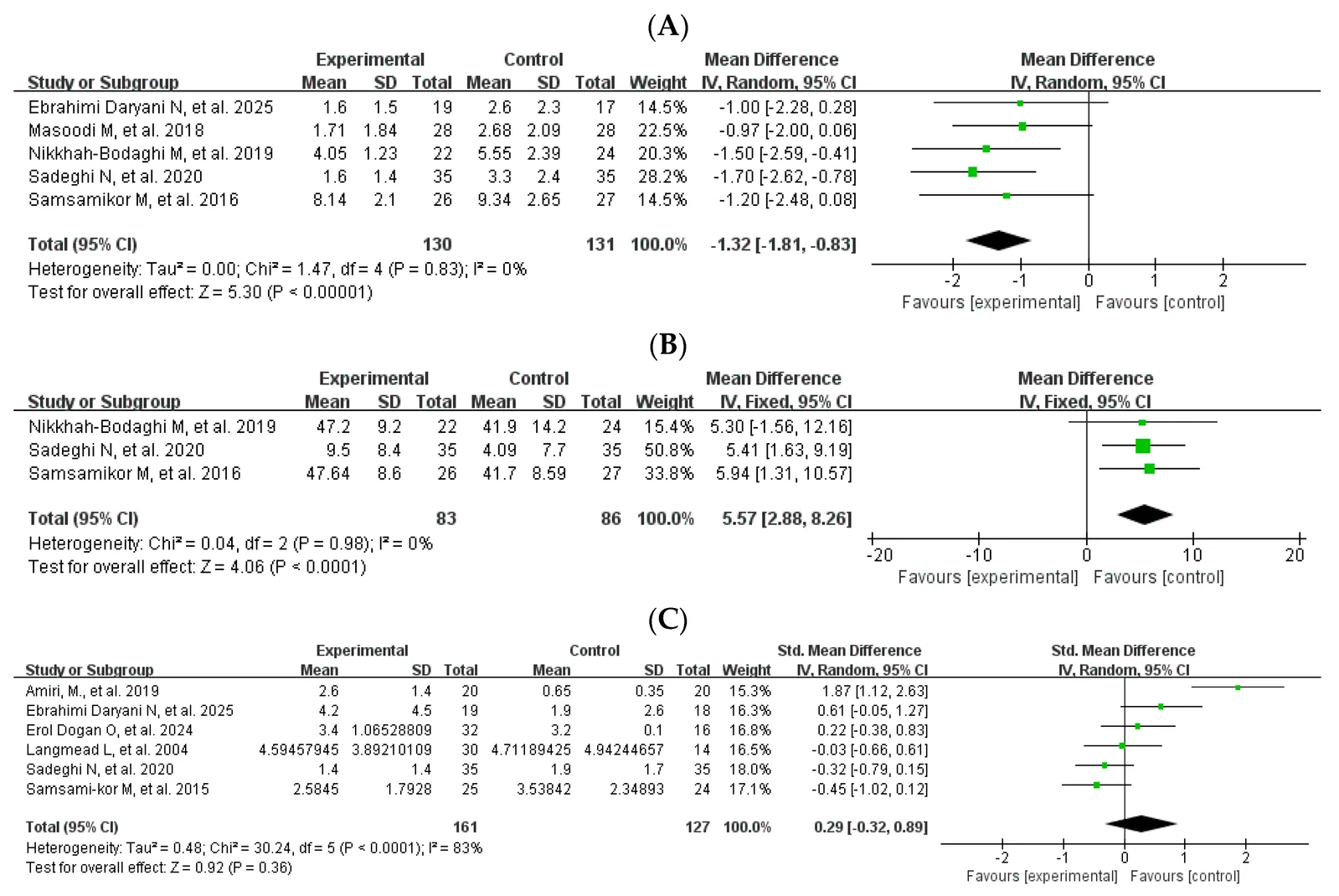
Disease activity, quality of life, and systemic inflammatory outcomes in conventional UC. (**A**) Post-intervention SCCAI. (**B**) IBDQ-9. (**C**) Post-intervention CRP/hs-CRP; in the three-arm Erol Dogan trial, the two active intervention arms were combined before comparison with the shared control [21,22,29,34,38,40,41,43,45]. Green squares represent individual study estimates, horizontal lines indicate 95% CIs, black diamonds denote pooled effects, and the vertical line indicates no effect.

**Figure 11:**
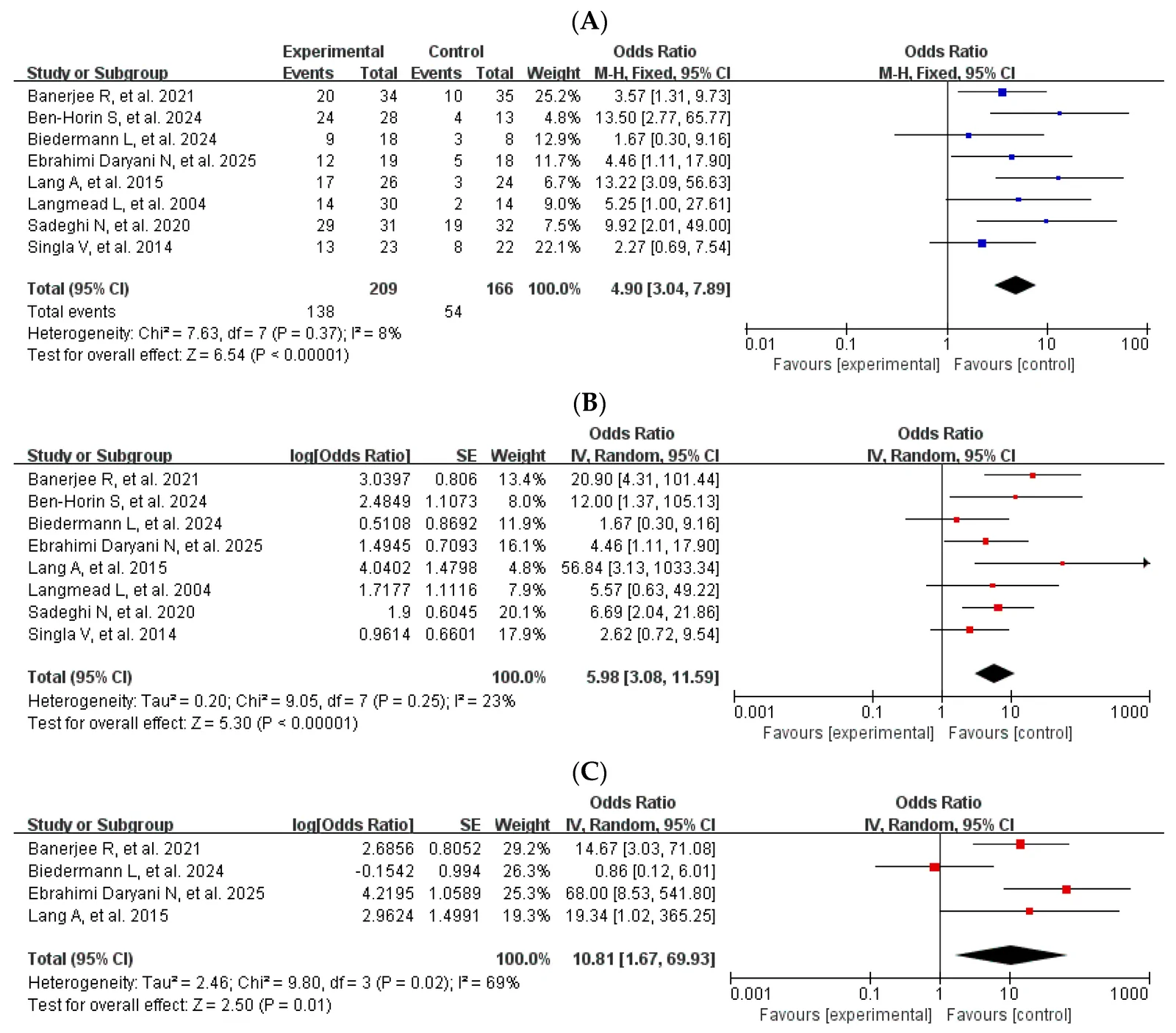
Key efficacy outcomes in conventional UC. (**A**) Clinical response. (**B**) Induction of clinical remission. (**C**) Strict endoscopic remission. Maintenance trials were excluded from panel (**B**), and endoscopic-improvement-only outcomes were excluded from panel (**C**) [20,30,34,35,36,40,42,45]. Colored squares represent individual study estimates, horizontal lines indicate 95% CIs, black diamonds denote pooled effects, and the vertical line at OR = 1 indicates no effect.

**Figure 12:**
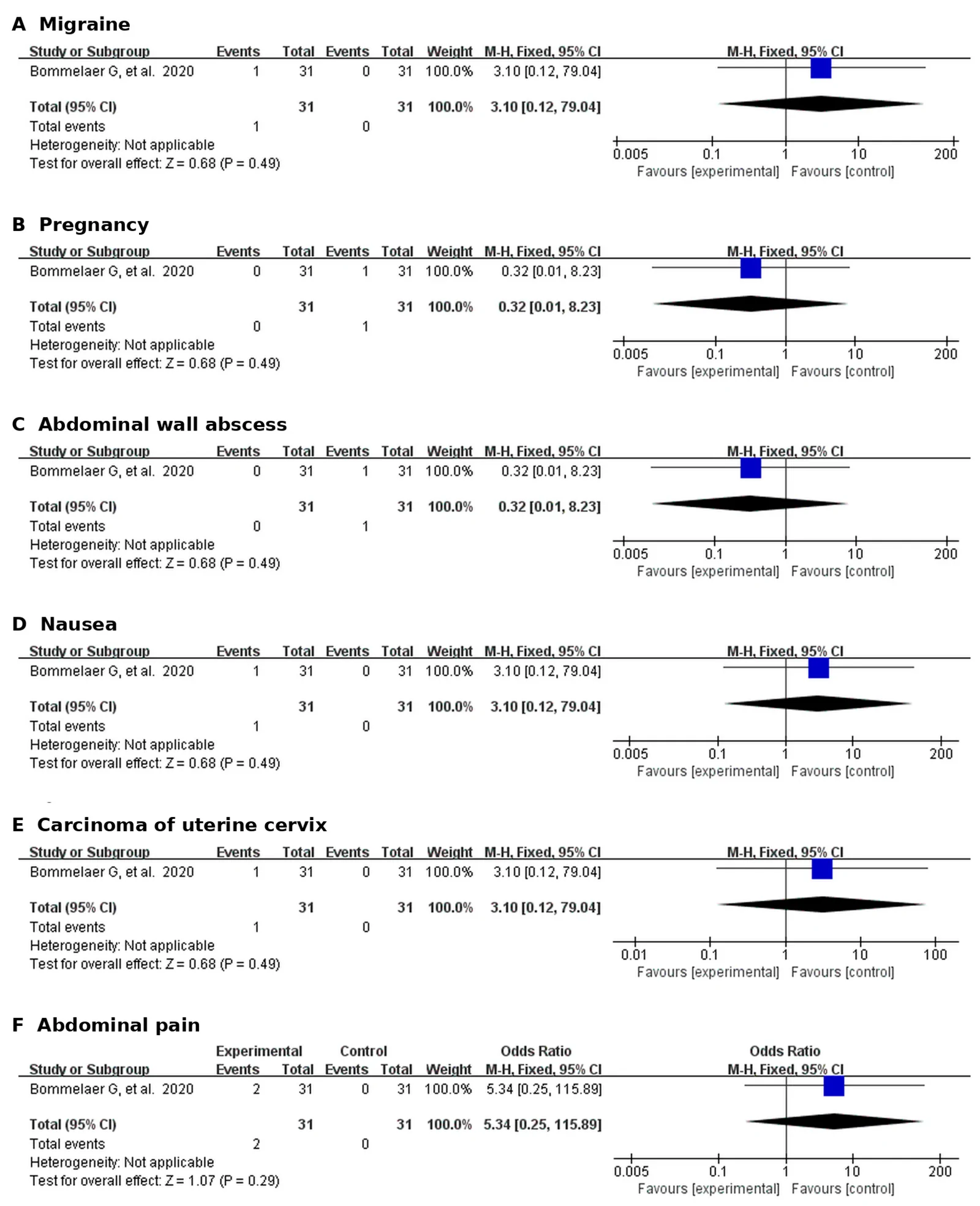
Event-specific serious adverse events in the Bommelaer et al. postoperative CD trial: (**A**) migraine; (**B**) pregnancy; (**C**) abdominal wall abscess; (**D**) nausea; (**E**) carcinoma of the uterine cervix; and (**F**) abdominal pain. Each category is displayed separately and is not pooled across events because all categories arose from the same randomized population [33]. Blue squares represent event-specific estimates, horizontal lines indicate 95% CIs, and the vertical line at OR = 1 indicates no effect.

**Figure 13:**
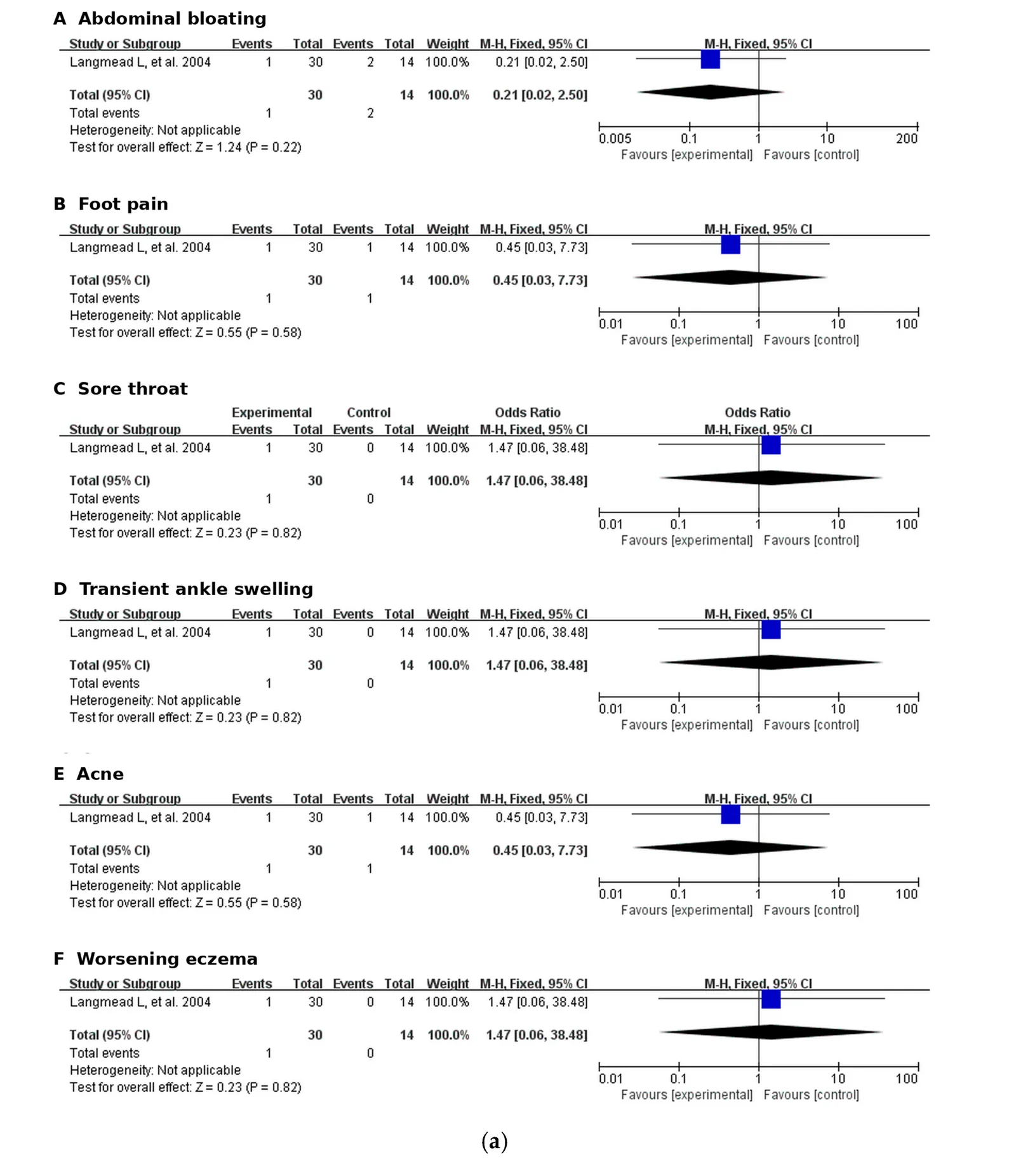

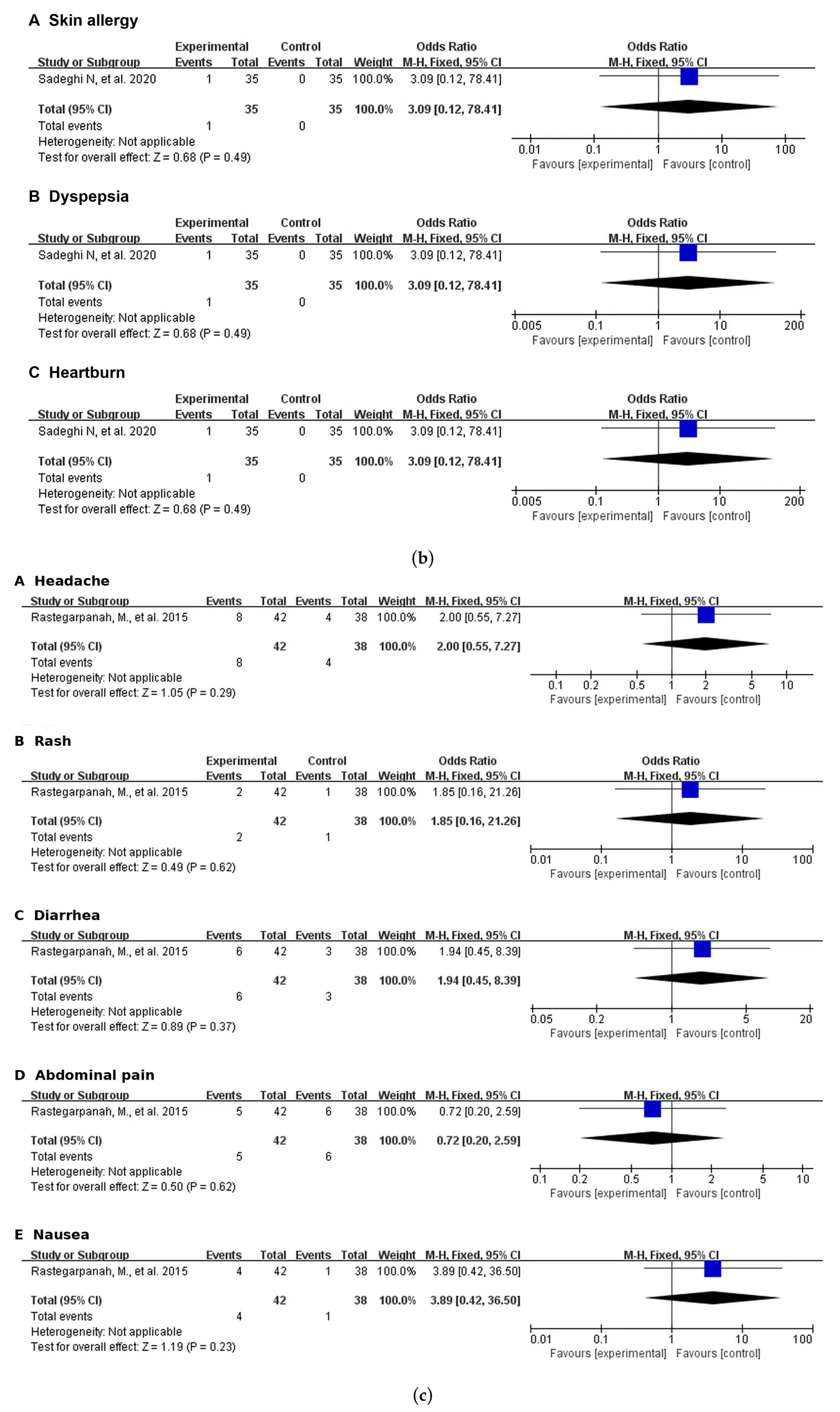

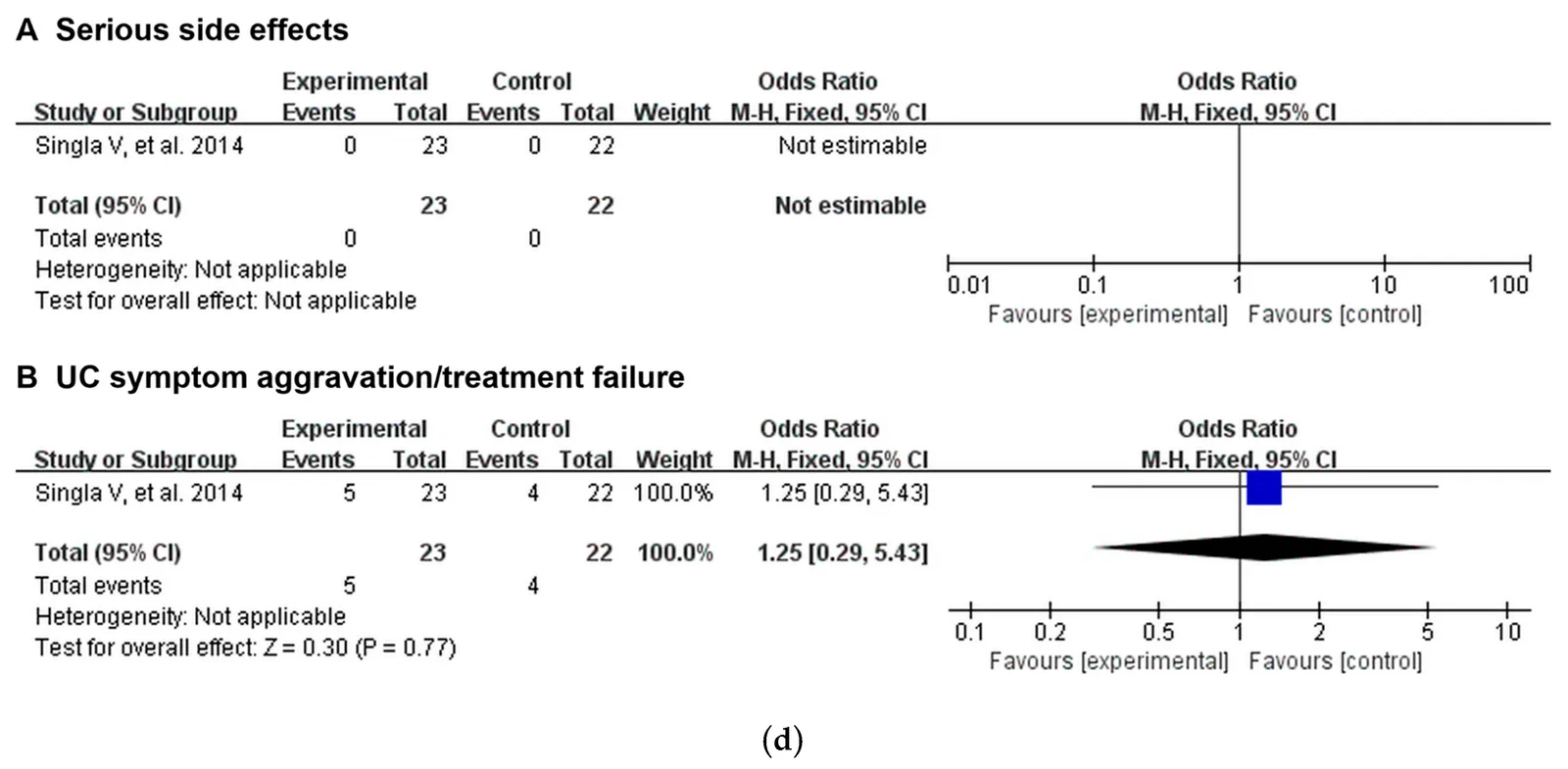
(**a**) Aloe vera trial by Langmead et al.: (A) abdominal bloating; (B) foot pain; (C) sore throat; (D) transient ankle swelling; (E) acne; and (F) worsening eczema. Event-specific adverse effects are displayed individually; no pooled cross-event effect is calculated. (**b**) Curcumin trial by Sadeghi et al.: (A) skin allergy; (B) dyspepsia; and (C) heartburn. All three events were reported in the same participant; the categories are shown separately and are not treated as independent patient-level events. (**c**) Silymarin trial by Rastegarpanah et al.: (A) headache; (B) rash; (C) diarrhea; (D) abdominal pain; and (E) nausea. The categories are displayed separately; because participant-level overlap was not reported, event counts are not summed into a composite endpoint. (**d**) Curcumin trial by Singla et al.: (A) serious side effects, with no events in either arm and therefore no estimable odds ratio; and (B) UC symptom aggravation/treatment failure (5/23 vs. 4/22), presented separately from adverse-event outcomes [36,40,44,45]. Blue squares represent event-specific estimates, horizontal lines indicate 95% CIs, the vertical line at OR = 1 indicates no effect.

**Figure 14:**
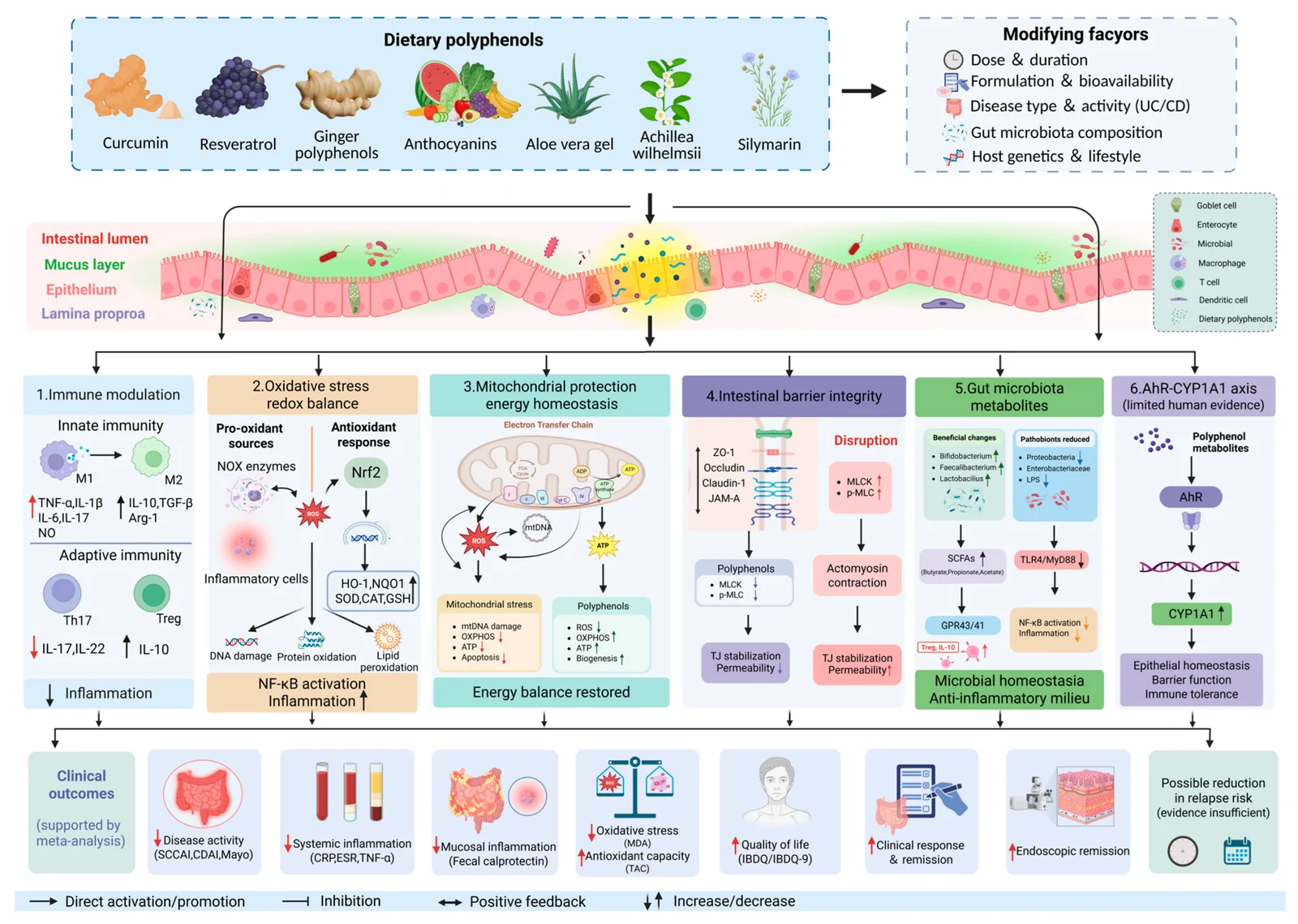
Integrative framework linking the clinical and biomarker findings of this review to the potential biological pathways of dietary polyphenols in IBD. The framework summarizes relationships between immune regulation, oxidative stress, mitochondrial function, epithelial barrier integrity, and microbial metabolism. The model is based empirically on clinical and biomarker results from the included randomized trials and the mechanistic literature is used to interpret and link these observations. The pathways therefore should be viewed as biologically plausible links that support interpretation of the clinical evidence rather than as individually established causal mechanisms. Arrow colors distinguish pathway categories and directional relationships; they do not represent quantitative effect sizes.

**Table 1:** Characteristics of included trials.

#	Polyphenol	Study	Country	Randomized Sample Size, *n*		Intervention		Relevant Outcomes	Mean Age, Years		Duration
				Intervention	Control	Intervention	Control		Intervention	Control	
1	Resveratrol	Samsamikor M et al. (2015) (2016) [21,29]	Iran	28	28	Resveratrol 500 mg/day orally for 6 weeks	Placebo for 6 weeks	SOD; TAC; MDA; SCCAI; IBDQ-9; between-group differences; disease activity; QoL	NR	NR	6 weeks
2	Resveratrol	Erol Dogan O et al. (2024) [22]	Türkiye	MD + curcumin 16 MD + resveratrol 16	16	MedDiet alone, MedDiet + curcumin 1600 mg/day, or MedDiet + resveratrol 500 mg/day for 8 weeks	The three arms served as mutual comparators	Disease activity; inflammatory markers; QoL; SF-36 pain domain; MEDAS score	NR	NR	8 weeks
3	Curcumin	Ben-Horin S et al. (2024) [30]	Israel; Greece	28	14	Enteric-coated CurQD 3 g/day (curcumin 1.5 g + QingDai 1.5 g) for 8 weeks, followed by curcumin 1.5 g/day for 8 weeks; stable background UC therapy	Enteric-coated placebo for 8 weeks; stable background UC therapy	Composite response; clinical remission; clinical response; endoscopic improvement; FC; AhR pathway; maintenance of response	Median 35 (IQR 23–48)	Median 25 (IQR 23–30)	16 weeks
4	Curcumin	Martins AS et al. (2024) (2025) [31,32]	Brazil	Curcumin 20 Curcumin + piperine 19	19	Curcumin 1000 mg/day or curcumin 1000 mg/day + piperine 10 mg/day for 12 weeks	Placebo for 12 weeks	SOD; FC; TNF-α; IL-17A; heartburn incidence	Curcumin 46.9 ± 18.9; curcumin + piperine 44.7 ± 12.4	50.9 ± 14.4	12 weeks
5	Curcumin	Bommelaer G et al. (2020) [33]	France	31	31	Curcumin 3 g/day (1 g tid) + thiopurine for 6 months	Placebo + thiopurine for 6 months	Postoperative endoscopic recurrence; clinical recurrence; IBDQ; serious AEs	35.0 ± 10.5	37.6 ± 13.8	6 months
6	Curcumin	Hanai H et al. (2006) [19]	Japan	45	44	Curcumin 2 g/day (1 g bid) + SZ 1.0–3.0 g/day or mesalamine 1.5–3.0 g/day for 6 months	Placebo + SZ/mesalamine for 6 months; background therapy continued for a further 6 months	Relapse rate; CAI; EI	45.2 ± 15.8	39.7 ± 14.2	6-month treatment + 6-month follow-up
7	Curcumin	Lang A et al. (2015) [20]	Israel; Hong Kong; Cyprus	26	24	Curcumin 3 g/day + optimized oral and topical mesalamine for 4 weeks	Placebo + optimized oral and topical mesalamine for 4 weeks	Clinical remission; clinical response; endoscopic remission	40.4 ± 12.7	41.4 ± 13.9	4 weeks
8	Curcumin	Ebrahimi Daryani N et al. (2025) [34]	Iran	21	22	SinaCurcumin^®^ 80 mg bid + mesalamine for 4 weeks	Placebo + mesalamine for 4 weeks	Clinical response; clinical remission; endoscopic remission; FC; IL-17; CRP; ESR; TNF-α	34.38 ± 8.7	34.58 ± 8.2	4 weeks
9	Anthocyanins	Biedermann L et al. (2024) [35]	Switzerland (6 centers)	24	10	Bilberry anthocyanin extract 800–1000 mg tid + background UC therapy for 8 weeks	Placebo + background UC therapy for 8 weeks	Clinical response; FC; endoscopic outcomes	NR	NR	8 weeks
10	Curcumin	Singla V et al. (2014) [36]	India	23	22	NCB-02 curcumin enema 140 mg/20 mL nightly + oral mesalamine 800 mg bid for 8 weeks	Placebo enema nightly + oral mesalamine 800 mg bid for 8 weeks	Clinical response; clinical remission; endoscopic improvement	32.7 ± 8.9	35.5 ± 13.8	8 weeks
11	Curcumin	Sugimoto K et al. (2020) [37]	Japan	20	10	Theracurmin^®^ 360 mg/day orally for 12 weeks	Placebo bid for 12 weeks	Clinical remission; CDAI; SES-CD; endoscopic remission; perianal lesions; AEs	36.3 ± 8.9	32.9 ± 13.4	12 weeks
12	Curcumin	Sadeghi N et al. (2020) [45]	Iran	35	35	Curcumin 500 mg tid (1500 mg/day) orally for 8 weeks	Placebo tid for 8 weeks	SCCAI; clinical response; clinical remission; hs-CRP; ESR	40.1 ± 13.2	40.6 ± 12.4	8 weeks
13	Curcumin	Masoodi M et al. (2018) [38]	Iran	29	29	Curcumin 80 mg tid (240 mg/day) + mesalamine 3 g/day for 4 weeks	Placebo + mesalamine 3 g/day for 4 weeks	SCCAI; defecation urgency; self-rated health; overall symptoms; AEs	38.21 ± 16.37	36.04 ± 11.78	4 weeks
14	Curcumin	He X et al. (2025) [39]	China	Maintenance 19 Induction 6	Maintenance 20 Induction 7	Curcumin + probiotics for 8 weeks (maintenance cohort *n* = 39; induction cohort *n* = 13)	Placebo + probiotics for 8 weeks	PDAI-based remission/response; post-IPAA pouchitis; excluded from conventional UC/IBD pooled analyses	NR	NR	8 weeks
15	Aloe vera	Langmead L et al. (2004) [40]	United Kingdom	30	14 ^b^	Oral aloe vera gel for 4 weeks	Placebo for 4 weeks	Clinical remission; clinical improvement; clinical response; SCCAI; histological score; sigmoidoscopy score; laboratory markers; AEs	Median 40 (22–76)	Median 36 [20,21,22,23,24,25,26,27,28,29,30,31,32,33,34,35,36,37,38,39,40,41,42,43,44,45,46,47,48,49,50,51,52,53,54,55]	4 weeks
16	Ginger	Nikkhah-Bodaghi M et al. (2019) [41]	Iran	32	32	Ginger powder 2000 mg/day orally for 12 weeks	Placebo for 12 weeks	MDA; SCCAI; IBDQ; TAC; oxidative-stress markers	41.41 ± 11.4	39.21 ± 11.81	12 weeks
17	Curcumin	Banerjee R et al. (2021) [42]	India	34	35	Bioenhanced curcumin (SMEDDS) 50 mg bid + mesalamine for 3 months, followed by 12-month follow-up	Placebo + mesalamine for 3 months, followed by 12-month follow-up	Clinical remission; clinical response; endoscopic remission	Median 38	Median 38	3-month treatment + 12-month follow-up
18	*Achillea wilhelmsii*	Amiri M et al. (2019) [43]	Iran	23	26	A. wilhelmsii 250 mg bid for 4 weeks	Placebo (HPMC capsules) for 4 weeks	Stool frequency; rectal bleeding; PGA; partial Mayo score; Hb; platelet count; ESR; CRP	34.82 ± 10.68	34.26 ± 10.21	4 weeks
19	Silymarin	Rastegarpanah M et al. (2015) [44]	Iran	42	38	Silymarin 140 mg once daily for 6 months	Placebo for 6 months	DAI; ESR; Hb; relapse rate; AEs	35.46 ± 3.64	36.81 ± 3.37	6 months

**Note:** Sample sizes in the main columns refer to the numbers randomized to each study arm whenever arm-level allocation was reported. Numbers completing follow-up or contributing to a specific outcome may be smaller because of attrition, missing outcome data, or per-protocol/available-case analyses; exact outcome-specific denominators are shown in the corresponding forest plots. For example, Rastegarpanah et al. randomized 42 participants to silymarin and 38 to placebo, of whom 38 and 32, respectively, completed the 6-month follow-up. Companion publications from the same underlying trial were counted once. (A) Martins et al. was a three-arm trial with 58 randomized participants. Arm-specific counts were reported inconsistently across the primary and secondary reports; the table therefore uses the internally consistent allocation of 20/19/19 (curcumin/curcumin + piperine/placebo). (B) Langmead et al. randomized 49 participants in total, but the complete randomized allocation by arm was not reported; the evaluable population was 30/14 and 24/11 completed follow-up. Values are mean ± standard deviation (SD) or median (interquartile range [IQR]) unless stated otherwise. Reported *p* values compare groups, and the intervention arm precedes the comparator throughout. **Abbreviations**: AE, adverse event; AhR, aryl hydrocarbon receptor; bid, twice daily; BMI, body mass index; CAI, clinical activity index; CD, Crohn’s disease; CDAI, Crohn’s Disease Activity Index; COVID-19, coronavirus disease 2019; D, follow-up duration; CRP, C-reactive protein; CurQD, curcumin–QingDai; DAI, disease activity index; EI, endoscopic index; ESR, erythrocyte sedimentation rate; FC, fecal calprotectin; FFM, fat-free mass; Hb, hemoglobin; HGS, handgrip strength; HPMC, hydroxypropyl methylcellulose; hs-CRP, high-sensitivity C-reactive protein; IBDQ, Inflammatory Bowel Disease Questionnaire; IBDQ-9, 9-item Inflammatory Bowel Disease Questionnaire; IL, interleukin; IQR, interquartile range; ITT, intention-to-treat; MD, Mediterranean diet; MDA, malondialdehyde; MEDAS, Mediterranean Diet Adherence Screener; NF-κB, nuclear factor kappa B; NR, not reported; OR, odds ratio; PDAI, Pouchitis Disease Activity Index; PGA, physician global assessment; POR, postoperative recurrence; PP, per-protocol; QoL, quality of life; SCCAI, Simple Clinical Colitis Activity Index; SES-CD, Simple Endoscopic Score for Crohn’s Disease; SF-36, 36-Item Short Form Health Survey; SMEDDS, self-microemulsifying drug delivery system; SOD, superoxide dismutase; SZ, sulfasalazine; TAC, total antioxidant capacity; tid, three times daily; TNF-α, tumor necrosis factor alpha; UC, ulcerative colitis.

**Table 2:** Interpretation of clinical endpoints in relation to proposed mechanisms.

Evidence Level	Measures Used in This Review	Permitted Interpretation	Interpretive Boundary
Patient report/clinical activity	SCCAI; total Mayo; partial Mayo; IBDQ	Possible change in symptoms, activity, or daily function	Does not by itself demonstrate mucosal healing or durable disease modification
Systemic inflammation	CRP/hs-CRP; ESR; TNF-α	Evidence of altered systemic inflammatory burden	Does not identify the responsible pathway or prove control of local mucosal disease
Mucosal inflammation	Fecal calprotectin	Evidence of lower neutrophil-associated intestinal inflammation	Cannot substitute for endoscopic or histological assessment
Oxidative stress	MDA; TAC; SOD	Evidence relevant to lipid peroxidation or redox balance	Does not prove Nrf2 activation, mitochondrial rescue, or another specific mechanism
Clinical/endoscopic outcome	Clinical response; clinical remission; endoscopic remission	Direct evidence of patient benefit or progress toward treatment targets	Sparse events and non-uniform definitions may enlarge ORs; long-term benefit is not established
Mechanistic inference	NF-κB; Nrf2; AhR; epithelial junctions; microbial metabolism	Biological context for interpreting the clinical pattern	Except for limited human surrogate data, this meta-analysis does not establish causal mediation

## Data Availability

No new primary data were generated. Data analyzed in this review were extracted from the published studies cited in the article. The complete database search strategies are described in Supplementary Table S1.

## Supplementary Materials

The following supporting information can be downloaded at https://www.mdpi.com/article/10.3390/nu18203335/s1. Table S1. Search Strategies for PubMed and Embase; Table S2. Trial-level Risk of Bias 2 assessment; Table S3. Sensitivity analyses excluding results at high risk because of missing outcome data; Table S4. Cutaneous or hypersensitivity-related adverse events; Table S5. Silymarin maintenance of remission: completer and intention-to-treat sensitivity analyses; Figure S1. Curcumin-specific disease-activity and inflammatory outcomes; Figure S2. Curcumin-specific study-level Mayo and recurrence outcomes; Figure S3. Exploratory IBD-wide biomarker and quality-of-life outcomes; Figure S4. Secondary disease-activity and inflammatory outcomes in conventional UC; Figure S5. Secondary inflammatory, redox, and hematologic outcomes in conventional UC; Figure S6. Additional clinical outcomes in UC; Figure S7. Disease- and setting-specific outcomes presented at the study level; Figure S8. Sensitivity analyses for sparse-event and missing-data concerns.
