# Supplementary material for: Dietary Polyphenols as Adjunctive Therapy for Inflammatory Bowel Disease: A Systematic Review and Meta-Analysis of Randomized Controlled Trials
Source: Nutrients. 2026 Oct 12;18(20):3335. doi: 10.3390/nu18203335 (PMC13708110; doi:10.3390/nu18203335)
Supplement: Supplementary file 1 [file nutrients-18-03335-s001.zip › nutrients-4586244-supplementary.pdf]

Supplementary Tables, Figures, Risk-of-Bias Assessment, and Sensitivity Analyses

Dietary Polyphenols as Adjunctive Therapy for Inflammatory Bowel Disease: A Systematic Review and Meta-analysis of Randomized Controlled Trials

The Supplement includes the complete database search strategies (Supplementary Table S1), trial-level RoB 2 domain judgments (Supplementary Table S2), sensitivity analyses excluding results at high risk because of missing outcome data (Supplementary Table S3), study-level cutaneous/hypersensitivity adverse-event data (Supplementary Table S4), silymarin completer and intention-to-treat sensitivity results (Supplementary Table S5), and Supplementary Figures S1-S8. When quantitative pooling was not considered clinically or methodologically appropriate, results are presented as individual-study estimates or descriptive summaries.

Supplementary Table S1. Search Strategies for PubMed and Embase

**PubMed**	(("Polyphenols"[Mesh] OR polyphenol*[tiab] OR "dietary polyphenol*"[tiab] OR flavonoid*[tiab] OR "phenolic compound*"[tiab] OR (Curcumin[tiab] OR Curcumas[tiab] OR Tumeric[tiab] OR Tumerics[tiab] OR Turmeric[tiab] OR Turmerics[tiab] OR "Curcuma longa"[tiab] OR "Curcuma zedoaria"[tiab]) OR (Resveratrol[tiab] OR Resveratrols[tiab] OR "3,4',5-stilbenetriol"[tiab] OR "3,5,4'-trihydroxystilbene"[tiab] OR "trans-resveratrol"[tiab] OR "cis-resveratrol"[tiab] OR "resveratrol-3-sulfate"[tiab]) OR (Quercetin[tiab] OR Quercetins[tiab]) OR (Naringenin[tiab] OR "naringenin-7-sulfate"[tiab]) OR (Anthocyanin*[tiab] OR Anthocyanidin*[tiab] OR Leucoanthocyanidin*[tiab]) OR (Hesperidin[tiab] OR Hesperetin[tiab] OR "Hesperetin-7-Rutinoside"[tiab]) OR (Catechin*[tiab] OR Epicatechin*[tiab] OR Epigallocatechin[tiab] OR "epigallocatechin gallate"[tiab] OR EGCG[tiab]) OR (Isoflavone*[tiab] OR Genistein[tiab] OR Daidzein[tiab]) OR (Silymarin[tiab] OR Silimarin[tiab]) OR (Proanthocyanidin*[tiab] OR Procyanidin*[tiab] OR "green tea polyphenol*"[tiab] OR "tea polyphenol*"[tiab] OR "grape seed polyphenol*"[tiab] OR "grape seed proanthocyanidin*"[tiab]) OR ("ellagic acid"[tiab] OR "gallic acid"[tiab])))<br>AND<br>("Inflammatory Bowel Diseases"[Mesh] OR "inflammatory bowel disease"[tiab] OR "inflammatory bowel diseases"[tiab] OR IBD[tiab] OR "Colitis, Ulcerative"[Mesh] OR "ulcerative colitis"[tiab] OR "Crohn Disease"[Mesh] OR "Crohn disease"[tiab] OR "Crohn's disease"[tiab] OR "Crohns disease"[tiab])<br>AND<br>(randomized controlled trial[pt] OR controlled clinical trial[pt] OR randomized[tiab] OR randomised[tiab] OR placebo[tiab] OR "drug therapy"[sh] OR random*[tiab] OR trial*[tiab] OR group*[tiab])<br>NOT<br>(animals[mh] NOT humans[mh])
**EMBASE**	('polyphenol'/exp OR polyphenol*:ti,ab OR 'dietary polyphenol*':ti,ab OR flavonoid*:ti,ab OR 'phenolic compound*':ti,ab)<br>OR<br>('curcumin'/exp OR curcumin:ti,ab OR curcuma*:ti,ab OR turmeric:ti,ab OR 'curcuma longa':ti,ab OR 'curcuma zedoaria':ti,ab)<br>OR<br>('resveratrol'/exp OR resveratrol*:ti,ab OR 'trans resveratrol':ti,ab OR 'cis resveratrol':ti,ab)<br>OR<br>('quercetin'/exp OR quercetin*:ti,ab)<br>OR<br>('naringenin'/exp OR naringenin*:ti,ab)<br>OR<br>('anthocyanin'/exp OR anthocyanin*:ti,ab OR anthocyanidin*:ti,ab OR leucoanthocyanidin*:ti,ab)<br>OR<br>('hesperidin'/exp OR hesperidin:ti,ab OR hesperetin:ti,ab)<br>OR<br>('catechin'/exp OR catechin*:ti,ab OR epicatechin*:ti,ab OR epigallocatechin:ti,ab OR 'epigallocatechin gallate':ti,ab OR EGCG:ti,ab)<br>OR<br>('isoflavone'/exp OR isoflavone*:ti,ab OR genistein:ti,ab OR daidzein:ti,ab)<br>OR<br>('silymarin'/exp OR silymarin:ti,ab OR silimarin:ti,ab)<br>OR<br>('proanthocyanidin'/exp OR proanthocyanidin*:ti,ab OR procyanidin*:ti,ab OR 'green tea polyphenol*':ti,ab OR 'tea polyphenol*':ti,ab OR 'grape seed polyphenol*':ti,ab OR 'grape seed proanthocyanidin*':ti,ab OR 'ellagic acid':ti,ab OR 'gallic acid':ti,ab)<br>AND<br>('inflammatory bowel disease'/exp OR 'inflammatory bowel disease':ti,ab OR 'inflammatory bowel diseases':ti,ab OR IBD:ti,ab OR 'ulcerative colitis'/exp OR 'ulcerative colitis':ti,ab OR 'crohn disease'/exp OR 'crohn disease':ti,ab OR 'crohn''s disease':ti,ab OR 'crohns disease':ti,ab)

	AND<br>('randomized controlled trial'/exp OR 'controlled clinical trial'/exp OR random*:ti,ab OR randomized:ti,ab OR randomised:ti,ab OR placebo*:ti,ab OR trial*:ti,ab OR group*:ti,ab)<br>NOT<br>('animal experiment'/exp NOT 'human experiment'/exp)

Supplementary Table S2. Trial-level Risk of Bias 2 assessment

RoB 2 is result-specific. Companion publications were linked at the trial level. For the Samsami-kor/Samsamikor resveratrol trial, the Domain 3 judgment differed by result (low for the 2015 inflammatory-marker report and some concerns for the 2016 oxidative-stress report); the overall trial-level classification remained some concerns. For the Martins AS companion reports, both assessed results were high risk overall because of missing outcome data. After linking companion reports, 19 independent randomized trials were represented: 4 low risk (21.1%), 9 with some concerns (47.4%), and 6 high risk (31.6%).

**Trial / linked reports**	**Representative result(s) assessed**	**D1**	**D2**	**D3**	**D4**	**D5**	**Overall**	**Key missing-data / analysis-set basis**
Samsami-kor / Samsamikor trial (2015/2016)	Inflammatory markers / oxidative-stress outcomes	Some concerns	Low	Low / Some concerns*	Low	Some concerns	Some concerns	Companion reports from the same resveratrol trial. D3 differed by result: low for the 2015 inflammatory-marker report and some concerns for the 2016 oxidative-stress report.
Erol Dogan 2024	Disease activity / CRP	Some concerns	Some concerns	Low	Some concerns	Some concerns	Some concerns	No high-risk missing-outcome signal in the RoB 2 workbook; some concerns relate mainly to randomization/blinding/reporting domains.
Ben-Horin 2024	Combined clinical + objective response	Low	Low	Low	Low	Low	Low	Randomized, blinded trial with prespecified outcome and no important missing-outcome concern in the assessed result.
Martins AS trial (2024/2025 companion reports)	Biomarker / body-composition outcomes	Low	Some concerns	High	Low	Some concerns	High	58 randomized. The biomarker report states 51 completed (14 placebo, 20 curcumin, 17 curcumin+piperine), while its flow diagram lists 14/20/19 analyzed; the 2025 companion report explicitly used per-protocol analyses. Attrition was unequal across arms.
Bommelaer 2020	Postoperative endoscopic recurrence	Low	Low	Low	Low	Low	Low	Double-blind postoperative trial with no high-risk missing-outcome concern for the assessed recurrence result.
Hanai 2006	Maintenance / relapse	Low	Low	Low	Low	Some concerns	Some concerns	No high-risk missing-outcome judgment; residual concern relates to selective-reporting certainty in an older trial.
Lang 2015	Clinical remission / response	Some concerns	Low	Low	Low	Low	Some concerns	No high-risk D3 judgment for the assessed result; randomization detail was judged incompletely reported.
Ebrahimi Daryani 2025	Clinical remission / biomarkers	Low	Some concerns	Some concerns	Low	Some concerns	Some concerns	Some loss to follow-up and analysis-set uncertainty, but not sufficient for a high D3 judgment in the RoB 2 assessment.
Biedermann 2024	Clinical / endoscopic outcomes	Some concerns	Some concerns	High	Low	Low	High	34 randomized, but only 26 entered the per-protocol set (18 ACRE, 8 placebo). Five ACRE and two placebo participants terminated early and one ACRE participant was excluded for protocol violation; entire missed visits were not imputed.
Singla 2014	Clinical response / remission	Low	Low	Some concerns	Low	Low	Some concerns	Some missing-outcome concern remained, but an ITT analysis was available and D3 was not judged high.
Sugimoto 2020	Clinical / endoscopic remission	Low	Low	Some concerns	Low	Low	Some concerns	Some withdrawals occurred, but the remission analysis used randomized denominators; D3 was not judged high.
Sadeghi 2020	SCCAI / inflammatory markers	Low	Low	Some concerns	Low	Low	Some concerns	Several withdrawals occurred; both ITT and PP results were reported, leading to some concerns rather than high D3 risk.
Masoodi 2018	SCCAI / self-rated health	Some concerns	Low	Low	Low	Low	Some concerns	Complete follow-up for the assessed continuous outcomes; remaining concern related to randomization reporting rather

Trial / linked reports	Representative result(s) assessed	D1	D2	D3	D4	D5	Overall	Key missing-data / analysis-set basis
								than missing data.
He 2025	Pouchitis response / remission	Low	Low	Low	Low	Low	Low	No important missing-outcome concern in the assessed result.
Langmead 2004	Clinical remission / CRP	Low	Low	High	Low	Some concerns	High	49 were randomized; 5 were excluded from further analysis after randomization, leaving 44 evaluable. Of those, 6/30 aloe and 3/14 placebo participants withdrew. LOCF was used for missing week-4 data, but the analysis was not a full randomized-denominator ITT.
Nikkhah-Bodaghi 2019	SCCAI / MDA	Some concerns	Some concerns	High	Low	Low	High	64 participants entered at baseline, but only 46 completed (22 ginger, 24 control). Participants with relapse/hospitalization, poor adherence (>10% capsules remaining), or unwillingness to continue were excluded from analysis.
Banerjee 2021	Clinical / endoscopic remission	Low	Low	Low	Low	Low	Low	No important missing-outcome concern for the main remission time point in the assessed result.
Amiri 2019	Partial Mayo / CRP	Some concerns	Some concerns	High	Some concerns	Low	High	49 entered the trial; 9 were excluded after randomization (3 treatment, 6 placebo), including one rash-related withdrawal and eight voluntary discontinuations; 20 per arm completed and were analyzed.
Rastegarpanah 2015	Maintenance of remission	Some concerns	Some concerns	High	Low	Some concerns	High	80 randomized (42 silymarin, 38 placebo); 10 discontinued (4 silymarin because of nausea; 6 placebo because of flare-up/abdominal pain). Although the paper states ITT, efficacy tables use completer denominators 38/32.

Supplementary Table S3. Sensitivity analyses excluding results at high risk because of missing outcome data

Sensitivity analyses were undertaken for key outcomes when exclusion of Domain 3 high-risk results left sufficiently comparable data to support a meaningful pooled estimate. For single-trial maintenance evidence, a conservative intention-to-treat sensitivity analysis was used instead of a pooled analysis.

Outcome	Main analysis	D3 high-risk result(s) excluded	Sensitivity analysis	Interpretation	Conclusion
UC SCCAI	5 studies; MD -1.32 (-1.81 to -0.83); I^2=0%	Nikkhah-Bodaghi 2019	4 studies; MD -1.28 (-1.82 to -0.73); I^2=0%	Direction and magnitude remained similar	Robust
UC CRP/hs-CRP	6 studies; SMD 0.29 (-0.32 to 0.90); I^2=83.7%	Amiri 2019; Langmead 2004	4 studies; SMD -0.02 (-0.47 to 0.44); I^2=61.3%	The pooled effect remained null	Robust null finding
Overall active-IBD clinical remission	9 studies; OR 6.13 (3.31 to 11.38); I^2=15%	Biedermann 2024; Langmead 2004	7 studies; OR 7.36 (3.75 to 14.44); I^2=13.5%	Favorable direction and statistical significance persisted	Robust
UC clinical remission	8 studies; OR 5.98 (3.08 to 11.59); I^2=22.6%	Biedermann 2024; Langmead 2004	6 studies; OR 7.31 (3.46 to 15.43); I^2=25.5%	Favorable direction and statistical significance persisted	Robust
Overall active-IBD endoscopic remission	5 studies; OR 9.33 (1.93 to 45.04); I^2=60.4%	Biedermann 2024	4 studies; OR 19.75 (6.70 to 58.19); I^2=0%	Direction persisted; magnitude changed substantially	Direction robust; magnitude unstable
UC endoscopic remission	4 studies; OR 10.81 (1.67 to 69.93); I^2=69.4%	Biedermann 2024	3 studies; OR 24.62 (7.76 to 78.15); I^2=0%	Direction persisted; magnitude changed substantially	Direction robust; magnitude unstable
Exploratory MDA	3 studies; SMD -1.52 (-3.12 to 0.07); I^2=94.0%	Martins AS trial; Nikkhah-Bodaghi 2019	Only one eligible study remained; no sensitivity meta-analysis was performed	Insufficient studies after exclusion	Not meta-analyzable
Silymarin maintenance of remission	Completer analysis: 35/38 vs 21/32; OR 6.11 (1.53 to 24.45)	Rastegarpanah 2015 (high D3)	Conservative ITT sensitivity: 35/42 vs 21/38; OR 4.05 (1.44 to 11.37)	Effect attenuated but direction persisted	Single-trial sensitivity

Supplementary Table S4. Cutaneous or hypersensitivity-related adverse events

Study	Event	Polyphenol group	Control group	Study-level OR (95% CI)	Interpretation
Amiri M, et al. (2019) [43]	Skin rash / contact dermatitis	1/23	0/26	3.53 (0.14 to 91.09)	No clear difference; very imprecise
Rastegarpanah M, et al. (2015) [44]	Rash	2/42	1/38	1.85 (0.16 to 21.26)	No clear difference; very imprecise
Sadeghi N, et al. (2020) [45]	Allergic symptoms	1/35	0/35	3.09 (0.12 to 78.41)	No clear difference; very imprecise

Note: No pooled estimate was calculated. For study-level odds ratios with a zero cell, a 0.5 continuity correction was applied to all four cells of the study-specific 2×2 table.

Supplementary Table S5. Silymarin maintenance of remission: completer and intention-to-treat sensitivity analyses

Study	Analysis set	Silymarin maintained remission	Placebo maintained remission	OR (95% CI)	Handling of dropouts	Interpretation
Rastegarpanah M, et al. (2015) [44]	Completer analysis	35/38	21/32	6.11 (1.53 to 24.45)	Only participants completing 6-month follow-up	Favored silymarin; wide CI
Rastegarpanah M, et al. (2015) [44]	Conservative ITT sensitivity	35/42	21/38	4.05 (1.44 to 11.37)	All randomized dropouts counted as non-remission	Effect attenuated but remained favorable

Note: The two rows are alternative analyses of the same randomized trial and were not treated as independent studies or pooled.

Figure S1

Curcumin-specific disease-activity and inflammatory outcomes.

A.

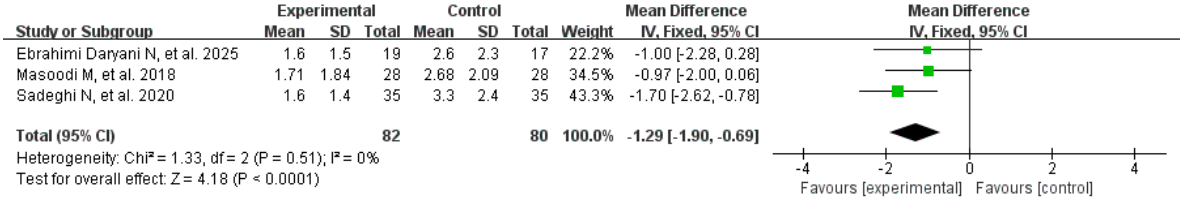

B.

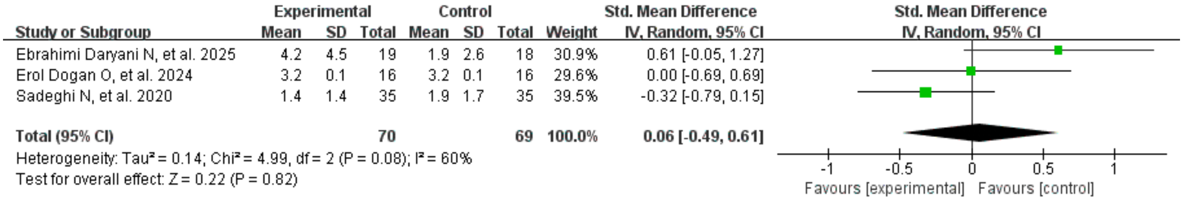

C.

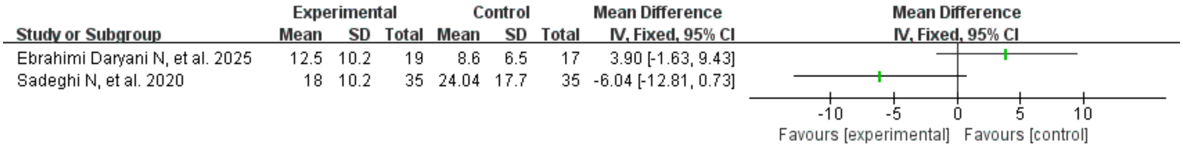

D.

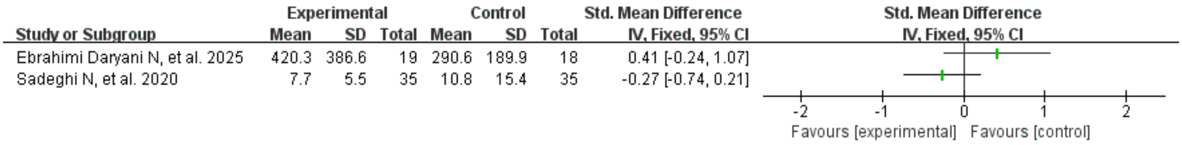

Figure S1. (A) SCCAI, pooled across three post-intervention datasets. (B) CRP/hs-CRP, pooled across three curcumin comparisons. (C) ESR and (D) TNF-α are presented as individual-study estimates because differences in study characteristics limit the interpretability of a combined estimate.

Figure S2

Curcumin-specific study-level Mayo and recurrence outcomes.

A.

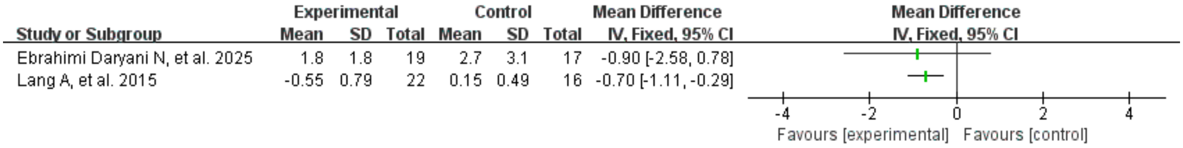

B.

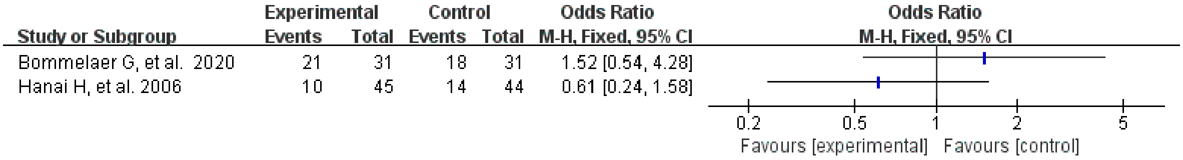

Figure S2. (A) Mayo-related outcomes are displayed without pooling because the studies used different Mayo constructs and data types. (B) Hanai evaluated UC maintenance relapse whereas Bommelaer evaluated postoperative CD recurrence; no common pooled estimand is assumed.

Figure S3

Exploratory IBD-wide biomarker and quality-of-life outcomes.

A.

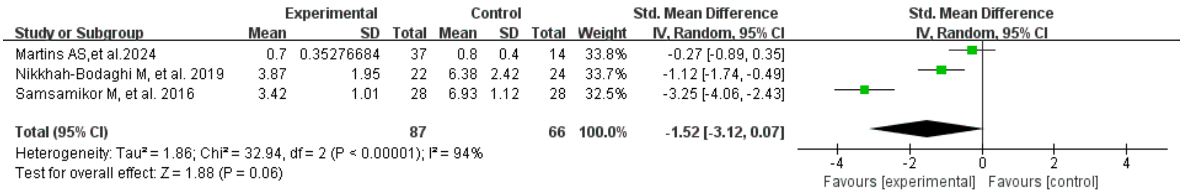

B.

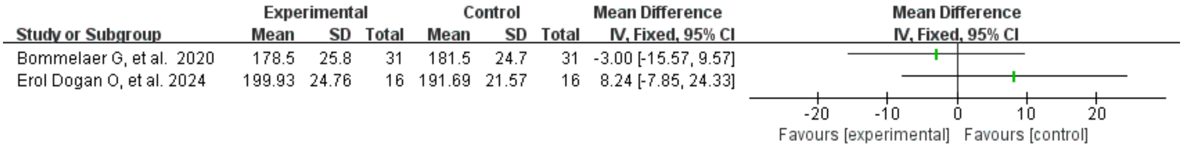

Figure S3. (A) Exploratory three-study MDA analysis; the two active arms of the three-arm Martins trial were combined before comparison with the shared placebo group. (B) Total IBDQ from two clinically different trials, shown without a pooled summary.

Figure S4

Secondary disease-activity and inflammatory outcomes in conventional UC.

A.

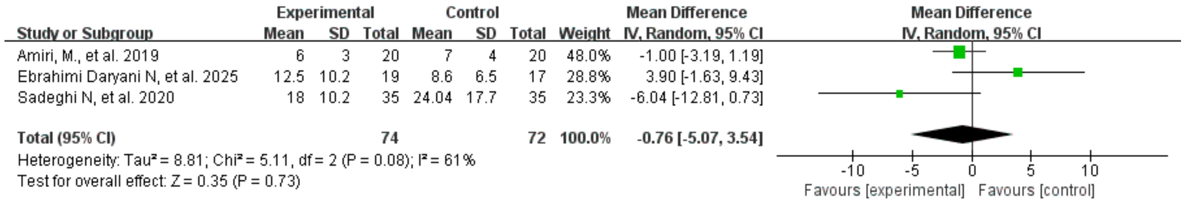

B.

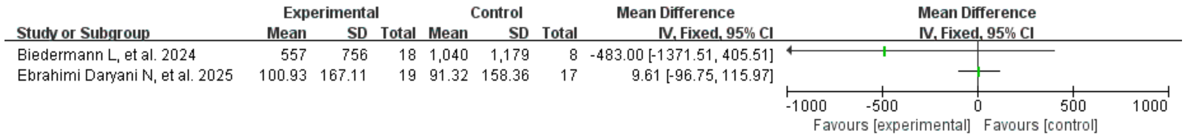

C.

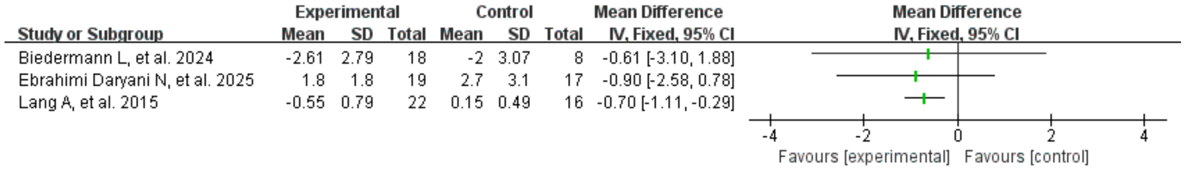

Figure S4. (A) ESR in active UC; Nikkhah-Bodaghi did not report ESR and the maintenance trial is presented separately. (B) Fecal calprotectin, shown as two individual study estimates. (C) Mayo-related continuous outcomes, shown without pooling because total Mayo, endoscopic Mayo subscore change, and partial Mayo change are distinct constructs.

Figure S5

Secondary inflammatory, redox, and hematologic outcomes in conventional UC.

A.

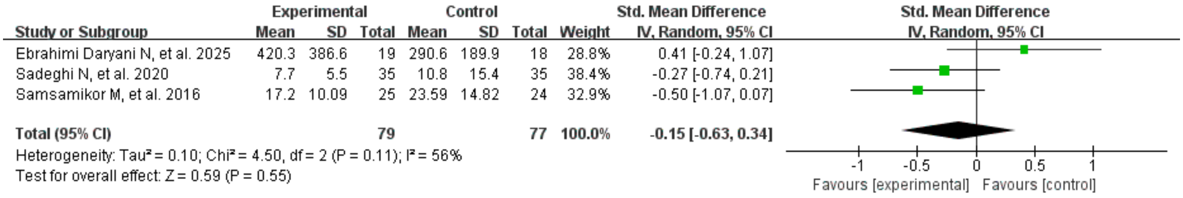

B.

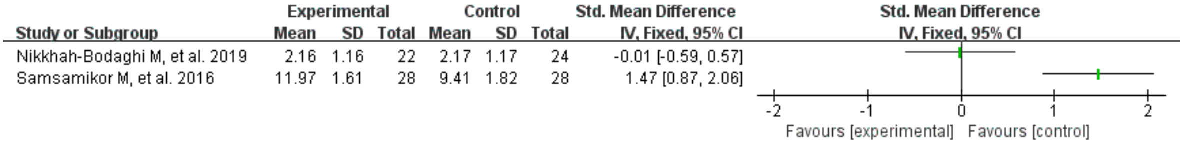

C.

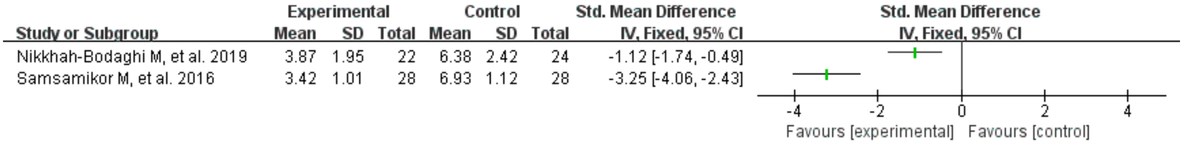

D.

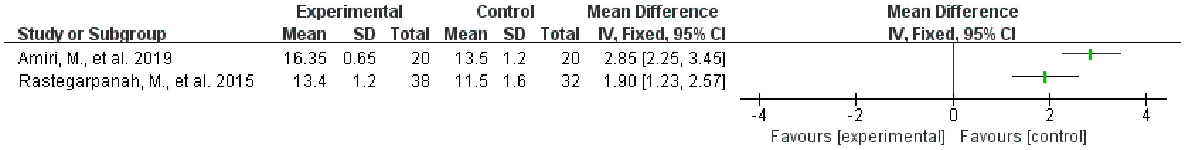

Figure S5. (A) TNF-α pooled across three compatible UC trials. (B) TAC, (C) MDA, and (D) hemoglobin are presented as study-level estimates where clinical or methodological differences limit meaningful quantitative synthesis.

Figure S6

Additional clinical outcomes in UC.

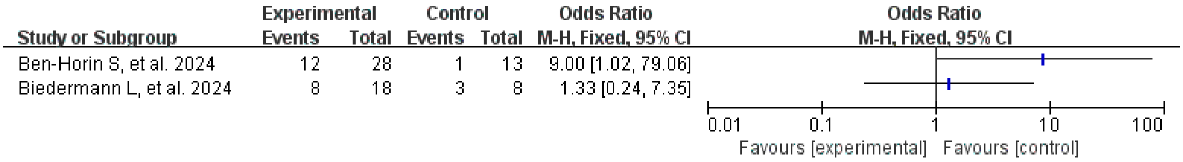

Figure S6. Distinct additional clinical outcomes shown as study-level effects: Biedermann symptomatic remission and Ben-Horin combined clinical-plus-objective response.

Figure S7

Disease- and setting-specific outcomes presented at the study level.

A.

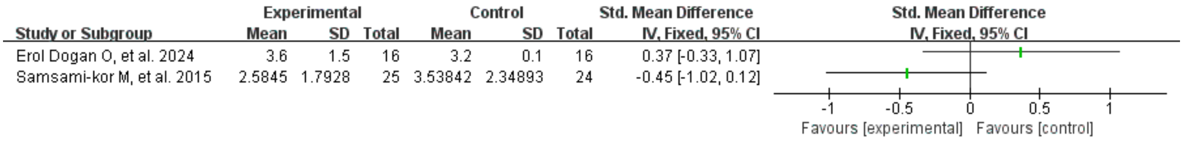

B.

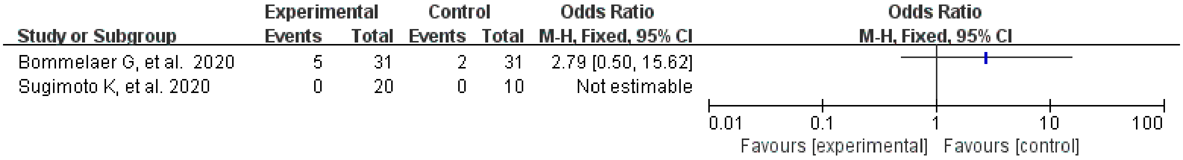

Figure S7. (A) Resveratrol CRP/hs-CRP using post-treatment values. (B) Serious adverse events in the CD trials. Results are presented at the individual-study level because differences in clinical setting and data characteristics limit the interpretability of pooled estimates.

Figure S8

Sensitivity analyses for sparse-event and missing-data concerns.

Study or Subgroup	Experimental Events	Experimental Total	Control Events	Control Total	Weight	Risk Difference M-H, Random, 95% CI
Banerjee R, et al. 2021	16	34	2	35	25.6%	0.41 [0.23, 0.60]
Ebrahimi Daryani N, et al. 2025	17	19	2	18	25.0%	0.78 [0.58, 0.98]
Lang A, et al. 2015	8	22	0	16	24.5%	0.36 [0.15, 0.58]
Sugimoto K, et al. 2020	3	20	0	10	24.9%	0.15 [-0.05, 0.35]
**Total (95% CI)**		**95**		**79**	**100.0%**	**0.43 [0.17, 0.68]**
Total events	44		4			

Heterogeneity: Tau² = 0.06; Chi² = 19.86, df = 3 (P = 0.0002); I² = 85%

Test for overall effect: Z = 3.27 (P = 0.001)

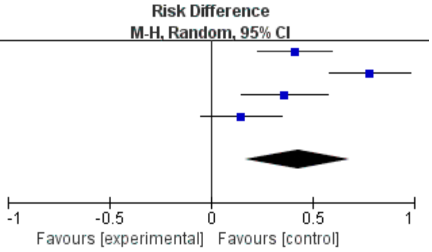

Figure S8. Risk-difference sensitivity analysis for curcumin endoscopic remission; this alternative scale does not require a continuity correction.
